# Ginsenoside Rh2-Pretreated Mesenchymal Stem Cell Exosomes Ameliorate Collagen-Induced Arthritis via N6-Methyladenosine Methylation

**DOI:** 10.34133/bmr.0220

**Published:** 2025-06-11

**Authors:** Zhongsheng Zhou, Shuhui Wu, Yang Li, Pu Shao, Jinlan Jiang

**Affiliations:** ^1^ Scientific Research Center, China-Japan Union Hospital of Jilin University, Changchun, China.; ^2^ Department of Orthopedics, China-Japan Union Hospital of Jilin University, Changchun, China.

## Abstract

This research examines the impact of exosomes derived from mesenchymal stem cells that have been pretreated with ginsenoside Rh2 (Rh2-pre Exo) in the context of collagen-induced arthritis (CIA). Rheumatoid arthritis (RA) is a persistent inflammatory condition marked by joint pain and swelling, which, in advanced stages, may result in joint damage and reduced functionality. We found that Rh2-pro Exo regulates the Toll-like receptor 4 (TLR4)/Myd88/nuclear factor κB (NF-κB) signaling pathway by modulating the m6A methylation levels of C-C motif chemokine receptor like 2 (CCRL2). The interaction between CCRL2 and TLR4 is a key factor influencing the activity of this signaling pathway. Our results indicate that this regulatory mechanism enhances the anti-inflammatory phenotype of macrophages, promoting a shift from pro-inflammatory to anti-inflammatory responses. Furthermore, treatment with Rh2-pre Exo substantially alleviated clinical symptoms and reduced joint damage in CIA models. These findings provide new insights into the therapeutic potential of Rh2-pre Exo in the treatment of RA, emphasizing the importance of m6A methylation in regulating immune responses. This study suggests that targeting the m6A methylation pathway of CCRL2 could offer a promising strategy for developing effective therapies for RA, ultimately improving patient outcomes and quality of life.

## Introduction

RA is an autoimmune disorder marked by ongoing inflammation, predominantly targeting the joints. This condition results in enduring pain, swelling, and a decline in functional ability [1]. As the disease progresses, RA can cause joint destruction, significantly impacting the quality of life for affected individuals [2]. Currently, treatment options for RA are diverse, including nonsteroidal anti-inflammatory drugs, glucocorticoids, and biological agents; however, the efficacy and safety of these therapies still have certain limitations [3]. Therefore, exploring new therapeutic strategies is particularly important.

Recent research has highlighted mesenchymal stem cells (MSCs) as a key area of interest in the treatment of RA because of their abilities to modulate the immune response and promote tissue repair [4,5]. Exosomes derived from MSCs are considered important mediators of their therapeutic effects, capable of regulating immune responses and promoting tissue repair through various mechanisms [6]. In addition to the exosomes themselves, engineering strategies for exosomes have also garnered significant attention, primarily including the following aspects.

First, gene editing techniques (such as CRISPR/Cas9) are employed to modify the exosome donor cells to enhance the specific functions of exosomes, such as inserting particular proteins or miRNAs, thereby improving their targeting and bioactivity [7,8]. Second, chemical or biological modifications to the surface of exosomes are utilized to enhance their stability and penetration ability, with common methods including liposomal encapsulation, polymer coating, and ligand binding [9–11]. Furthermore, loading drugs, RNA, or other biomolecules into exosomes as drug delivery carriers can enhance the bioavailability and targeting of therapeutics, thereby improving efficacy [12–15]. These strategies expand the application potential of engineered exosomes in areas such as cancer therapy, regenerative medicine, and immunotherapy.

Furthermore, reducing the oxygen levels during cell culture or adding drugs and cytokines can induce cells to release exosomes with specific functions [16–18]. This approach helps enhance the anti-inflammatory, antitumor, or regenerative properties of the exosomes. Ginsenoside Rh2 is a natural compound known for its anti-inflammatory and immunomodulatory effects [19]. Our preliminary research has found that the combined use of ginsenoside Rh2 and MSC exosomes can improve collagen-induced arthritis (CIA) symptoms in rats by modulating the gut microbiota and its metabolites [20]. However, it remains unclear whether ginsenoside Rh2-preconditioned MSC exosomes can alleviate CIA symptoms.

Macrophage polarization is a critical factor in the development of RA [21]. In RA, macrophages can polarize into M1 or M2 types, each affecting inflammation and joint damage differently [22]. M1 macrophages exhibit pro-inflammatory properties and are usually activated by pathogens, cytokines [including interferon-γ (IFN-γ)], and Toll-like receptor (TLR) stimulation. They generate significant quantities of pro-inflammatory cytokines such as tumor necrosis factor-α (TNF-α), interleukin-1β (IL-1β), and IL-6, along with nitric oxide (NO) [23]. This promotes inflammation in the early stages of RA, leading to joint swelling and pain. In contrast, M2 macrophages possess anti-inflammatory and tissue repair functions, generally active during wound healing and the resolution of inflammation [24]. The TLR4/Myd88/NF-κB signaling pathway plays a critical role in macrophage polarization [25]. TLR4, functioning as a pattern recognition receptor, identifies pathogen-associated molecular patterns (PAMPs) and damage-associated molecular patterns (DAMPs), thereby triggering an immune response [26]. Upon TLR4 activation, downstream signaling molecules are recruited via the Myd88 adapter protein, activating the NF-κB transcription factor [27]. In macrophages, TLR4 activation typically leads to polarization toward the M1 type (pro-inflammatory) [25]. Consequently, modulating the TLR4/Myd88/NF-κB signaling pathway to suppress M1 macrophage polarization could serve as an effective approach for the treatment of RA.

N6-methyladenosine (m6A) methylation is the most prevalent posttranscriptional modification of mRNA, primarily found in eukaryotic cells [28]. The in-depth exploration of m6A modifications has opened new avenues for posttranscriptional gene regulation in eukaryotes [29]. The m6A modification process primarily entails the involvement of methyltransferases (commonly referred to as Writers), such as METTL3, METTL14, and WTAP; demethylases (known as Erasers) like FTO and ALKBH; and methylation readers (designated as Readers), including YTHDF1, YTHDF2, and YTHDF3 [30]. In recent years, m6A modifications have garnered widespread attention for their roles in gene expression and immune response regulation. Research indicates that m6A methylation plays a crucial role in the pathogenesis of RA [31]. Specifically, m6A methylation influences the expression of inflammation-related genes by regulating mRNA stability, translation efficiency, and degradation [32]. In RA, the expression of specific pro-inflammatory cytokines (such as IL-6 and TNF-α) is modulated by m6A methylation, thereby affecting macrophage polarization and activity [33,34]. The overexpression of these cytokines is closely linked to the inflammatory response in RA. However, whether Rh2-pro Exo can regulate m6A methylation levels remains to be further investigated.

This research seeks to investigate the mechanism of action of Rh2-pre Exo in a CIA model. The results of the cellular experiments indicate that Rh2-pre Exo effectively inhibits the proliferation and migration of M1 macrophages while also suppressing M1 macrophage polarization and promoting M2 macrophage polarization. The in vivo study results show that Rh2-pre Exo similarly inhibits M1 macrophage polarization and promotes M2 macrophage polarization, thereby substantially improving arthritis symptoms. Additionally, our proteomic sequencing findings suggest that the TLR4/Myd88/NF-κB pathway may play a pivotal role in this process. Importantly, combined methylation sequencing and mRNA sequencing results show that, compared to the arthritis group, the m6A levels of C-C motif chemokine receptor like 2 (CCRL2) mRNA in the synovium of mice treated with Rh2-pre Exo were significantly elevated. We verified the interaction between CCRL2 and TLR4. Integrating proteomic data, methylation sequencing results, and other experimental findings, we hypothesize that Rh2-pro Exo may improve CIA by modulating the m6A methylation levels of CCRL2, thereby affecting the TLR4/Myd88/NF-κB pathway.

## Materials and Methods

### Materials

The following reagents and kits were used: Cell Counting Kit-8 (CCK-8) Assay Kit (Beyotime, Shanghai, China), IL-1β, IL-6, IL-10, and TNF-α enzyme-linked immunosorbent assay (ELISA) Kits (Nanjing Jincheng Institute of Biotechnology, China), 4′,6-diamidino-2-phenylindole (DAPI) (Beyotime, Shanghai, China), SF555-phalloidin (Solarbio, Beijing, China), Cy5.5 (TOPSCIENCE, Shanghai, China), 3,3′-dioctadecyloxacarbocyanine perchlorate (DIO) (Beyotime, Shanghai, China), Masson’s Trichrome Stain Kit (Solarbio, Beijing, China), Modified Saffron-O and Fast Green Stain Kit (Solarbio, Beijing, China), MTX (Solarbio, Beijing, China), Rh2 [≥97.0% high-performance liquid chromatography (HPLC), Sigma, Germany], Click-iT EdU-555 Imaging Kit (Thermo Fisher Scientific, China), lipopolysaccharide (LPS) (Solarbio, Beijing, China), Protein A+G Agarose Beads (P2108, Beyotime, Shanghai, China), TRIzol Reagent (Takara, Japan), PrimeScript RT Master Mix RR036A (Takara, Japan), SYBR Premix Ex Taq RR420A (Takara, Japan), BCA Assay Kit (Beyotime, Shanghai, China), Complete Freund’s Adjuvant (CFA) (Chondrex, USA), Incomplete Freund’s Adjuvant (IFA) (Chondrex, USA), Type II Bovine Collagen (Chondrex, USA), Adipogenic Differentiation Medium (Sigma, Germany), Osteogenic Differentiation Medium (Sigma, Germany), Chondrogenic Differentiation Medium (Sigma, Germany), Osteogenesis Assay Kit (Beyotime, Shanghai, China), Alcian Blue Staining Kit (Beyotime, Shanghai, China), and Oil Red O Staining Kit (Beyotime, Shanghai, China).

### Identification of MSCs

MSCs were cultured in Adipogenic Differentiation Medium (Sigma, Germany) for a period of 18 to 21 d, followed by staining with oil red (Beyotime, Shanghai, China) for 10 to 20 min. The resulting images were captured and analyzed using microscopy. For the osteogenic differentiation, MSCs were maintained in Osteogenic Differentiation Medium (Sigma, Germany) for 28 d and subsequently subjected to Alizarin Red staining (Beyotime, Shanghai, China) for 20 to 30 min. Microscopic observation and imaging occurred after this staining process. To promote chondrogenesis, MSCs were grown in Chondrogenic Differentiation Medium (Sigma, Germany) for 21 d to form chondrospheres. These sections were stained with Alcian Blue (Beyotime, Shanghai, China) for 30 min, rinsed 3 times with water, and then counterstained with Nuclear Solid Red for 5 min before being examined and photographed under a microscope. Flow cytometry was employed to assess the phenotypic characteristics of MSCs, utilizing antibodies against specific cell surface markers, including phycoerythrin (PE)-conjugated anti-human CD105, fluorescein isothiocyanate (FITC)-conjugated anti-human CD90 (Thy1), PE-conjugated anti-human CD73 (ecto-5’-nucleotidase), FITC-conjugated anti-human CD11b, FITC-conjugated anti-human CD19, FITC-conjugated anti-human CD34, PE-conjugated anti-human CD45, and PE-conjugated anti-human HLA-DR, all acquired from BioLegend, USA.

### MSC-exo isolation and identification

The human umbilical cord tissue was rinsed thoroughly 3 times with phosphate-buffered saline (PBS). Subsequently, the umbilical cord was dissected into small pieces and placed in a 100-mm culture dish containing 8 to 10 ml of Dulbecco’s modified Eagle’s medium (DMEM) enriched with 20% fetal bovine serum (FBS). The tissue fragments were arranged with a 3-mm gap between them. The dish was then incubated at 37 °C in a CO_2_ incubator, with medium changes occurring every 3 d. Once the surrounding cells achieved approximately 80% confluence, preparations for passaging began. The umbilical cord fragments were carefully removed along with the primary culture medium and washed twice with PBS. A digestive enzyme solution was applied to the dish to facilitate cell dissociation. To terminate the digestion process, fresh culture medium was added, and the cells were transferred into a centrifuge tube, where they were centrifuged at 1,000 rpm for 5 min. After discarding the supernatant, the cells were resuspended in T75 culture flasks and incubated at 37 °C with 5% CO_2_. Experiments were conducted using cells from the 3rd to 6th generations, and the supernatant from MSC cultures was collected. This supernatant underwent a series of centrifugation steps: first at 200*g* for 10 min to remove dead cells, followed by 2,000*g* for another 10 min to eliminate cellular debris. Then, centrifugation at 10,000*g* for 1 h was performed to isolate additional microvesicles, and a final centrifugation at 100,000*g* for 90 min was conducted to remove any contaminating proteins [35]. Exosomes were isolated by collecting the precipitate following the final centrifugation step. Nanoparticle tracking analysis (NTA; NanoSight LM10, UK) was employed to evaluate the size and concentration of the exosomes. Each sample underwent 5 analyses, while exosome morphology was investigated using an 80-kV transmission electron microscope (TEM; Hitachi, Japan). The concentration of exosomes for this study was calculated based on their protein content, which was measured using the bicinchoninic acid (BCA) assay.

### Western blot analysis

RAW264.7 cells were plated evenly in 6-well plates at a density of 400,000 cells per well and cultured overnight at 37 °C in a 5% CO₂ atmosphere to facilitate proper adhesion. Depending on the experimental groups, appropriate treatment solutions were added, and cells were incubated for an additional 24 h. After washing the cells 3 times with PBS, 100 μl of lysis buffer [radioimmunoprecipitation assay (RIPA):phenylmethylsulfonyl fluoride (PMSF), 100 mM in a 99:1 ratio, Abcam, USA] was introduced to each well to lyse the cells. The lysates were then centrifuged at 16,000*g* for 15 min to isolate protein samples.

For protein extraction from rat synovial tissue, 20 mg of the sample was weighed and washed 3 times with PBS. The sample was placed into a grinding tube, finely chopped using scissors, and then combined with 200 μl of lysis buffer (RIPA:PMSF, 100 mM in a 99:1 ratio, Abcam, USA). Homogenization was carried out at 4 °C with a tissue homogenizer, followed by a 20-min lysis period on ice. The resulting mixture was then transferred to a centrifuge and spun at 12,000 rpm for 20 min at 4 °C to separate the supernatant. The protein concentration was determined using the BCA assay.

Next, protein samples were combined with 5× protein loading buffer in a 4:1 ratio and denatured at 95 °C for 5 min. Following sodium dodecyl sulfate–polyacrylamide gel electrophoresis (SDS-PAGE) electrophoresis, the target proteins were transferred to a nitrocellulose (NC) membrane (Millipore). The membrane was blocked with 5% skim milk at room temperature for 1 h and then incubated overnight at 4 °C with primary antibodies specific to inducible nitric oxide synthase (INOS) (rabbit, 1:500, Proteintech, USA), ARG1 (rabbit, 1:10,000, Proteintech, USA), CD86 (mouse, 1:10,000, Santa Cruz Biotechnology, USA), CD206 (rabbit, 1:10,000, Abcam, USA), Myd88 (rabbit, 1:500, Proteintech, USA), NF-κB (rabbit, 1:5,000, Proteintech, USA), p-NF-κB (rabbit, 1:5,000, Proteintech, USA), ALKBH5 (rabbit, 1:5,000, Proteintech, USA), TLR4 (mouse, 1:5,000, Proteintech, USA), CCRL2 (mouse, 1:1,000, Abcam, USA), β-actin (mouse, 1:20,000, Proteintech, USA), β-tubulin (rabbit, 1:20,000, Proteintech, USA), and glyceraldehyde-3-phosphate dehydrogenase (GAPDH) (mouse, 1:20,000, Proteintech, USA).

After 3 washes with tris-buffered saline containing Tween 20 (TBST), the NC membrane was incubated at room temperature for 1 h with the appropriate secondary antibody (1:5,000, Proteintech, China). Following 3 more washes with TBST, the membrane was prepared for detection. Exosomes were characterized using the same protocol, utilizing antibodies against CD9 (rabbit, 1:1,000, Abcam, USA), TSG101 (rabbit, 1:1,000, Proteintech, China), and calnexin (rabbit, 1:1,000, Proteintech, China).

### Animal experiment design

Male DBA1/J mice (weighing 15 ± 2 g and aged 7 weeks) and male Sprague–Dawley (SD) rats (8 weeks old and weighing between 180 and 200 g) were procured from Beijing Weitong Lihua Laboratory Animal Technology Co. Ltd. (Beijing, China). The animals were housed in a controlled environment maintained at 25 °C and 60% humidity, with continuous access to food and water. All animal experiments received approval from the Jilin University Animal Experimentation Ethics Committee, ensuring that they adhered to established standards for animal care.

Prior to the experiments, the rats underwent a 1-week acclimatization period. Bovine type II collagen was prepared by dissolving it in a 1% glacial acetic acid solution, resulting in a 2 mg/ml solution, designated as liquid A. This was mixed in equal volumes with Freund’s complete adjuvant to form an emulsion, referred to as liquid B, for the primary immunization. Each animal received a subcutaneous injection of 100 μl of liquid B into the dorsal area of the tail root and the dorsum of the foot. Following a 14-d interval, secondary immunization was performed using liquid C, an emulsion of liquid A mixed with incomplete Freund’s adjuvant in a 1:1 ratio, yielding a final collagen concentration of 1 mg/ml. The model was typically established within 7 to 14 d [36].

In total, 32 animals were utilized to establish the CIA model. On day 21, successfully modeled rats were randomly assigned to 4 groups (6 rats per group): The model group was given 200 μl of PBS; the MSC-exo group received 500 μg/kg MSC-exo; the Rh2-pre Exo group was administered 500 μg/kg Rh2-pre Exo; and the MTX group was treated with 5 mg/kg MTX. All drugs were administered via tail vein injection. Six additional animals without modeling were used as the control group. Beginning on day 24, the animals were evaluated every 4 d using the arthritic index scores (AIS), following established protocols. The total arthritis score for all 4 limbs was obtained by summing the individual joint scores, with a maximum possible score of 16 for each animal. Paw thickness in the right lower limb was measured every 4 d using Vernier calipers.

### Immunocytochemistry procedure

After deparaffinization and rehydration of the tissue slides, we conducted antigen retrieval, followed by blocking of endogenous peroxidase and serum. The slides were then incubated overnight at 4 °C with primary antibodies (IL-1β, TNF-α, IL-6) at a dilution of 1:100. Following this incubation, secondary antibodies were applied at room temperature for 30 min. The slides were subsequently subjected to 3,3′-diaminobenzidine (DAB) staining and counterstained with hematoxylin. Finally, they were dehydrated, mounted, and analyzed under a microscope.

### Safranin O/Fast Green staining procedure

The Safranin O/Fast Green staining method, tailored for cartilage, facilitates the distinction of the 3 layers present within the osteochondral structure. The procedure begins with dewaxing knee joint sections in water, followed by rinsing with PBS solution. The sections are stained with Weigert staining solution for 3 to 5 min, differentiated in acidic ethanol for 15 s, and subsequently rinsed in distilled water for 1 min. Next, the sections are immersed in Fast Green staining solution for 5 min, followed by soaking in distilled water for an additional minute. They are then stained with Safranin O for 2 min and rinsed again in distilled water for 1 min. Subsequently, the sections are treated with acetic acid solution for 2 min, followed by another minute in distilled water. After a final wash with distilled water and another 2-min acetic acid treatment, the sections are dehydrated in ethanol, cleared with xylene, and mounted with neutral gum. The prepared sections are then analyzed and photographed using an optical microscope.

### Hematoxylin and eosin staining procedure

This staining technique is designed to visualize cellular structures and tissue morphology and involves several key steps. Initially, knee joints are fixed in 4% paraformaldehyde for 3 to 4 d. Following fixation, the joints are decalcified in a 10% EDTA solution until sufficient decalcification is achieved. After this, the joints are dehydrated. The next step is to embed the dehydrated joints in paraffin. Once embedded, the joints are histologically sectioned to a thickness of 5 μm. The sections are then subjected to hematoxylin and eosin (H&E) staining. The final step involves observing the stained sections under a light microscope for pathological evaluation.

### Micro-CT and x-ray examination of ankle specimens

Following the experiment, the animals were euthanized, and their lower limbs were dissected and fixed in 4% paraformaldehyde. The ankle joints and paws were examined using micro-CT (computed tomography) (SCANCO Medical AG, Switzerland) and x-ray (Bruker, Germany) with consistent parameters, focusing on the measurement and analysis of relevant bone parameters. The procedure commenced with a 3-dimensional reconstruction of the standard ankle specimen using micro-CT. Subsequently, 3-dimensional reconstruction images of each treatment group were obtained, providing a comprehensive view of structural changes. Relevant bone parameters were then measured and analyzed based on these images. The final step involved careful observation and assessment of the effects of each treatment group through the combined micro-CT and x-ray examinations. This dual approach offers detailed insights into structural alterations and bone parameters, enhancing the understanding of the effects of various treatments on ankle joint tissues.

### In vivo targeting capability test

To test the targeting ability of Rh2-pre Exo and MSC-exo in arthritic mice, we performed the following experiments: 10 μl of 1 mM Cy5.5 (Topscience, China) was added to 200 μl of PBS containing 100 μg of Rh2-pre Exo or MSC-exo, mixed thoroughly, and incubated at room temperature for 1 h. After incubation, unbound Cy5.5 was removed through ultracentrifugation, and the pellet was collected and resuspended in 500 μl of PBS.

The MSC-exo group was injected intravenously into CIA rats with 500 μg/kg of Cy5.5-labeled MSC-exo. The Rh2-pre Exo group received an intravenous injection of 500 μg/kg of Cy5.5-labeled Rh2-pre Exo, while the control group was administered an injection of 500 μl of Cy5.5 solution.

### Exosome uptake experiment

The experiment was divided into 2 groups, Rh2-pre Exo and MSC-exo. RAW264.7 cells were induced to differentiate into M1 macrophages using μg/ml LPS before the start of the experiment. Rh2-pre Exo and MSC-exo were initially labeled with DIO (Beyotime) and resuspended in PBS. Unbound DIO dye was removed by centrifuging the solution at 100,000*g* at 4 °C for 2 h, collecting the pellet containing the DIO-labeled exosomes. The concentration of exosomes was then determined using the BCA method.

DIO-labeled exosomes were incubated with cells from each experimental group at a concentration of 20 μg/ml for 4 h. Following 3 washes with PBS, the cells were fixed on ice using 4% paraformaldehyde for 20 min. After fixation, they were subjected to 3 additional washes with PBS and then permeabilized at room temperature for 10 min with 0.5% Triton X-100, followed by another 3 washes with PBS.

For the staining procedure, 2 μl of SF555 fluorescently labeled phalloidin stock solution (Solarbio) was diluted in 200 μl of PBS and added to the confocal dish, where the cells were incubated at room temperature for 20 min. After rinsing with PBS, the cells were fixed again with 4% paraformaldehyde for 20 min and stained with DAPI (Beyotime) for 10 min. Following a final wash with PBS, the cells were observed and captured using a confocal imaging system (Olympus FV1200).

### Macrophage proliferation and migration assays

#### Proliferation

MSCs were treated with varying concentrations of Rh2 (5, 10, 20, 40, and 80 μM), and cell viability was measured using the CCK-8 assay to identify the optimal concentration for pretreated stem cells. Then, exosomes derived from stem cells at different doses (1, 5, and 10 μg/ml) were cocultured with LPS-stimulated RAW264.7 cells, and quantitative polymerase chain reaction (qPCR) was used to assess the expression levels of INOS and ARG1 to determine the optimal exosome dose. Cell proliferation was evaluated using the CCK-8 (Beyotime). Specifically, 10,000 cells were plated in each well of a 96-well plate and incubated for 24 h in a 37 °C environment with 5% CO₂. After this incubation period, 10 μl of CCK-8 solution was added to each well and allowed to incubate in the dark for 1 h. The optical density (OD) was subsequently measured using a multifunctional microplate reader (Infinite200pro, Tecan, Switzerland). Cell viability was calculated using the following formula: Cell viability = [(Experimental well − Blank well)/(Control well − Blank well)] × 100%. Furthermore, 5-ethynyl-2′-deoxyuridine (EdU) labeling, fixation, and permeabilization were performed following the manufacturer’s guidelines with the Click-iT EdU-555 Imaging Kit (Thermo Fisher Scientific, China). Observations and imaging were conducted using a fluorescence microscope.

#### Migration

The migration ability of M1 macrophages was evaluated using the Transwell assay. A cell suspension of 2 × 10^4^ cells was diluted in 0.2 ml of serum-free culture medium and placed in the upper chamber of a Transwell (Corning). Treatments were applied according to the experimental groups, with LPS (1 μg/ml) used to induce M1 macrophages in RAW264.7 cells. Complete culture medium was added to the lower chamber. After 24 h, the upper layer of cells was removed, and the cells on the bottom surface were stained with crystal violet (Beyotime, China). Following PBS washes, stained cells were counted under an optical microscope.

### Flow cytometry

Cells from each treatment group were collected and washed twice with PBS. They were fixed in 4% paraformaldehyde at room temperature for 20 min and then centrifuged at 300*g* for 5 min to obtain the pellet. Subsequently, the cells underwent permeabilization at room temperature for 10 min using 1% Triton X-100, followed by another centrifugation at 300*g* for 5 min to collect the pellet once more.

To prevent nonspecific binding, the cells were placed on ice with 0.25 μg of anti-mouse CD16/32 (BioLegend, USA) per 10^6^ cells in 100 μl for a duration of 10 min. The cells were then incubated in the dark at 4 °C for 30 min with Brilliant Violet 421 anti-mouse F4/80 (BioLegend, USA), along with either allophycocyanin (APC) anti-mouse CD86 or PE anti-mouse CD206 (MMR) (both sourced from BioLegend, USA).

After one wash, the cells were resuspended in PBS and analyzed via flow cytometry. The flow cytometry data were subsequently processed using FlowJo software version 10.2 (BD, USA).

### Enzyme-linked immunosorbent assay

Synovial tissue samples from each group of mice were collected and rinsed 3 times with PBS. The tissues were then thoroughly homogenized on ice using a tissue homogenizer. Following homogenization, the samples were centrifuged at 3,000 rpm for 20 min at 4 °C to separate the supernatant. The concentrations of inflammatory mediators such as IL-1β, IL-6, IL-10, and TNF-α were measured using ELISA kits (Nanjing Jincheng Institute of Biotechnology, China). All procedures were conducted following the manufacturer’s guidelines rigorously.

### Immunofluorescence staining

#### Cell immunofluorescence

RAW264.7 cells were seeded evenly in confocal culture dishes and cultured until reaching approximately 70% confluence. After 24 h of treatment according to the different experimental groups, the cells were fixed with 4% paraformaldehyde for 20 min at room temperature. Following fixation, the cells underwent several washes with PBS and were then permeabilized using 0.5% Triton X-100 for 20 min. After additional PBS washes, the cells were blocked with 5% goat serum for 30 min. Primary antibodies (CD86, CD206, NF-κB) were diluted 1:200 and incubated overnight at 4 °C. After 3 PBS washes, fluorophore-conjugated secondary antibodies (1:500) were added and incubated for 1 h at room temperature. The cells were subsequently stained with DAPI for 5 min, washed with PBS, and then observed, imaged, and analyzed using a fluorescence microscope.

#### Tissue immunofluorescence

For tissue samples, dewaxing was performed using xylene, followed by rehydration. Antigen retrieval was accomplished by boiling the samples in citrate buffer at pH 6.0 for 10 min. Following this, the sections were washed with PBS and permeabilized with 0.3% Triton X-100 for 15 min. To block nonspecific binding, they were treated with 5% bovine serum albumin for 1 h. The paraffin-embedded sections were then incubated overnight at 4 °C with the primary antibodies. Afterward, the sections were washed with PBS and incubated at room temperature for 1 h with fluorophore-conjugated secondary antibodies, ensuring that they were shielded from light. Finally, the samples were stained with DAPI for 20 min and then rinsed with PBS.

### Hemolysis assay

Fresh anticoagulated rat blood (10 ml) was collected into a centrifuge tube and centrifuged at 1,500 rpm for 10 min. The supernatant was discarded, and the resulting pellet of red blood cells was washed 3 times with PBS until the supernatant became colorless. After the final wash, 100 μl of red blood cells was added to the Rh2-pre Exo solution to achieve final concentrations of 1, 5, 10, and 20 μg/ml. Positive and negative control groups were included in the experiment. The positive control group consisted of deionized water, and the negative control group consisted of PBS solution.

### Protein extraction and trypsin digestion

The protein extraction process commenced by grinding the sample into a fine powder with liquid nitrogen, which was then transferred to a 5-ml centrifuge tube. Four volumes of lysis buffer containing 1% Triton X-100 and a 1% protease inhibitor cocktail were introduced, followed by sonication on ice for 3 min using a high-intensity ultrasonic processor (Scientz). For experiments involving posttranslational modifications (PTMs), specific inhibitors were incorporated into the lysis buffer, such as 3 μM trichostatin A (TSA) and 50 mM nicotinamide (NAM) for acetylation, along with a 1% phosphatase inhibitor for phosphorylation. After mixing, debris was eliminated through centrifugation at 12,000*g* for 10 min at 4 °C, and the protein concentration in the supernatant was assessed using a BCA kit. To precipitate proteins, trichloroacetic acid (TCA) was added to achieve a final concentration of 20% (m/v), followed by vortexing and incubation for 2 h at 4 °C. The precipitate was then collected via centrifugation at 4,500*g* for 5 min at 4 °C, washed 3 times with precooled acetone, and dried. Subsequently, the protein sample was reconstituted in 200 mM triethylammonium bicarbonate (TEAB) and dispersed ultrasonically. Trypsin was added at a 1:50 mass ratio of trypsin to protein for overnight digestion. The sample was reduced with 5 mM dithiothreitol for 30 min at 56 °C and alkylated with 11 mM iodoacetamide for 15 min at room temperature in the dark, and finally, the peptides were desalted using a Strata X SPE column.

### Liquid chromatography-tandem mass spectrometry analysis

The tryptic peptides were reconstituted in solvent A and subsequently loaded onto a custom-made reversed-phase analytical column measuring 25 cm in length with a 100 μm inner diameter. The mobile phase comprised solvent A (which included 0.1% formic acid and 2% acetonitrile in water) and solvent B (0.1% formic acid in acetonitrile). Peptide separation was performed using the following gradient: from 0 to 14 min, solvent B increased from 6% to 24%; from 14 to 16 min, it further rose from 24% to 35%; from 16 to 18 min, it surged from 35% to 80%; and from 18 to 20 min, it stabilized at 80% B, all at a consistent flow rate of 500 nl/min on a NanoElute UHPLC system (Bruker Daltonics). Ionization of the peptides was achieved using a capillary source, and they were analyzed with a timsTOF Pro 2 mass spectrometer, which operated at an electrospray voltage of 1.75 kV. The time-of-flight (TOF) detector was utilized to identify both precursor and fragment ions in data-independent parallel accumulation serial fragmentation (dia-PASEF) mode. The full mass scan range was set between 300 and 1,500 m/z, with 20 PASEF-tandem mass spectrometry (MS/MS) scans collected for each cycle. The MS/MS scan range was confined to 400 to 850 m/z, featuring an isolation window of 7 m/z.

### Protein annotation and functional enrichment

Gene Ontology (GO) analysis was conducted utilizing eggnog-mapper software to extract GO IDs from identified proteins within the EggNOG database. This process classified the proteins according to their cellular components, molecular functions, and biological processes. For Kyoto Encyclopedia of Genes and Genomes (KEGG) pathway annotation, protein identification was performed through BLAST comparisons (blastp, *e* value ≤ 1 × 10^−4^), with annotations derived from the top-scoring sequences in the KEGG database. Subcellular localization predictions were executed using Wolf PSORT for general proteins and PSORTb for the subcellular structures of prokaryotic proteins. For clusters of orthologous groups (COG)/eukaryotic orthologous groups (KOG) annotation, proteins were categorized into prokaryotic (COG) or eukaryotic (KOG) classifications. Compared to the COG database from NCBI, EggNOG provides a more extensive classification of species and homologous protein sequences, including phylogenetic tree construction and functional annotation for each homologous gene cluster. To perform hierarchical clustering based on the functional classifications of differentially expressed proteins (e.g., GO, Domain, and KEGG pathway), we first gathered the categories obtained from enrichment analysis, along with their corresponding *P* values. Categories were filtered to include only those enriched in at least one cluster with a *P* value of <0.05. The resulting *P* value matrix was then transformed using *x* = −log10 (*P* value). These transformed *P* values were clustered using one-way hierarchical clustering (Euclidean distance, average linkage) in Genesis. Finally, cluster memberships were visualized through a heatmap generated using the “Heatmap” function from the “ComplexHeatmap” R package.

### Parallel reaction monitoring validation

The resulting MS data were analyzed using Skyline (v.3.6), quantifying each protein based on 2 unique peptides. The peptide settings were adjusted as follows: The enzyme was specified as trypsin [KR/P], allowing for a maximum of 2 missed cleavages. Peptide length was limited to between 8 and 25 residues, and variable modifications included carbamidomethyl on cysteine and oxidation on methionine, with up to 3 variable modifications permitted.

Transition settings were established with precursor charges of 2 and 3, ion charges of 1 and 2, and included ion types *b*, *y*, and *p*. Product ions were defined starting from ion 3 to the last ion, with an ion match tolerance set at 0.02 Da.

### MeRIP-seq for whole transcriptome RNA methylation (m6A sequencing)

The RNA methylation sequencing method, designed for low-input samples, is an enhancement of the conventional m6A-seq approach, specifically addressing the removal of trace rRNA. Initially, deoxyribonuclease (DNase) I is used to eliminate genomic DNA (gDNA) from total RNA. All methylation-modified fragments of coding and noncoding RNAs are then enriched using m6A-specific antibodies. Following this, the Takara micro-scale library preparation kit (SMARTer Stranded Total RNA-Seq Kit v2) is employed to selectively remove rRNA-derived molecules from the library and construct the library. High-throughput sequencing and data analysis are subsequently performed, focusing primarily on mRNA and lncRNA, enabling the detection of m6A modification sites across all expressed RNAs in specific tissues under defined conditions.

### Molecular docking

Protein searches were conducted using the Protein Data Bank (PDB) and UniProt databases, filtering by species, chain length, and resolution to download the target protein PDB file. Structural analysis of protein interactions was performed using AlphaFold3, yielding interaction conformations and scoring, from which dominant conformations were selected and output as files [37]. The dominant conformation PDB files were uploaded to the PDBePISA website to predict relevant data on protein interaction forces, interacting amino acid residues, interaction surface area, and binding energy. Based on the analysis results from PDBePISA, visualization of interacting amino acids was carried out using PyMOL (v. 2.5.5) and Discovery Studio Client, which included annotating bond lengths and changing visualization colors to distinguish different types of interactions, such as hydrogen bonds and salt bridges. Visualization files were then generated for output.

### RNA dot blot assay

Samples with known concentrations were gradient-diluted in enzyme-free centrifuge tubes. The RNA samples were heated at 95 °C for 3 min and then immediately transferred to ice for rapid cooling to prevent the reformation of secondary structures. A 2-μl aliquot of each RNA sample was carefully spotted onto a nitrocellulose (NC) membrane in sequence. The membrane was left at room temperature to air-dry slightly before being transferred to a 37 °C incubator for 30 min. Afterward, the membrane was washed with an appropriate amount of TBST for 5 min to remove unbound RNA. Following the wash, TBST was discarded, and the membrane was incubated with a blocking solution at room temperature with shaking for 1 h. After removing the blocking solution, m6A antibody (mouse/immunoglobulin G3 (IgG3), 1:1,000, Proteintech) diluted in TBST was added, and the membrane was incubated overnight at 4 °C. The membrane was then washed 3 times with TBST, each wash lasting 5 min. After discarding TBST, the membrane was incubated with a TBST-diluted secondary antibody for 1 h at room temperature. The secondary antibody was then removed, and the membrane was washed 3 times with TBST for 5 min each. The membrane was incubated in enhanced chemiluminesence (ECL) detection reagent for 2 min, and images were captured using an ECL chemiluminescence imager. After imaging, the membrane was incubated in methylene blue staining buffer with gentle shaking at room temperature for 30 min. The membrane was rinsed with distilled water until the background was clear, which took approximately 60 s, and photographed again.

### Co-immunoprecipitation analysis

Mouse synovial tissue was lysed using NP-40 lysis buffer (Beyotime, Shanghai, China) supplemented with a protease inhibitor cocktail (Sigma-Aldrich) to ensure protein integrity. Specific immunoprecipitation of CCRL2 was achieved by incubating the lysates with an immunoprecipitation-grade TLR4 antibody (rabbit/IgG, 1:1,000, Proteintech) at 4 °C. In parallel, an isotype control antibody (rabbit/IgG, 1:1,000, Proteintech) was used for the control group. The antibody–protein complexes were subsequently incubated with protein A+G agarose beads (P2108, Beyotime, Shanghai, China) for 4 h at 4 °C. Afterward, the immunoprecipitated proteins were eluted from the beads, followed by analysis via Western blotting (WB).

### Real-time fluorescence quantitative PCR analysis

Total RNA extraction was carried out using TRIzol reagent (Takara, Japan), followed by reverse transcription with PrimeScript RT Master Mix RR036A (Takara, Japan). Primer sequences were designed and synthesized by Sangon Biotech (see Table S1 for details). Real-time fluorescence quantitative PCR (RT-qPCR) was conducted using SYBR Premix Ex Taq RR420A (Takara, Japan). The relative expression levels of the target genes were determined using the 2^−ΔΔCt^ method, normalizing against GAPDH as the control. The resulting data were then compared to the normalized control values.

### Statistical analysis

Statistical analysis was performed using GraphPad Prism 9.0 (GraphPad Software Inc., USA). Normality tests were conducted to evaluate the distribution of continuous variables, which informed the choice of statistical methods. When the data exhibited a normal distribution, parametric tests, including Student’s *t* test or one-way analysis of variance (ANOVA), were employed. In cases where the data did not meet parametric assumptions, nonparametric tests, such as the Wilcoxon rank sum test or Kruskal–Wallis *H* test, were utilized. A *P* value of less than 0.05 was regarded as statistically significant.

## Results

### Characterization of MSC and Rh2-pre Exo

**Fig. 1.**
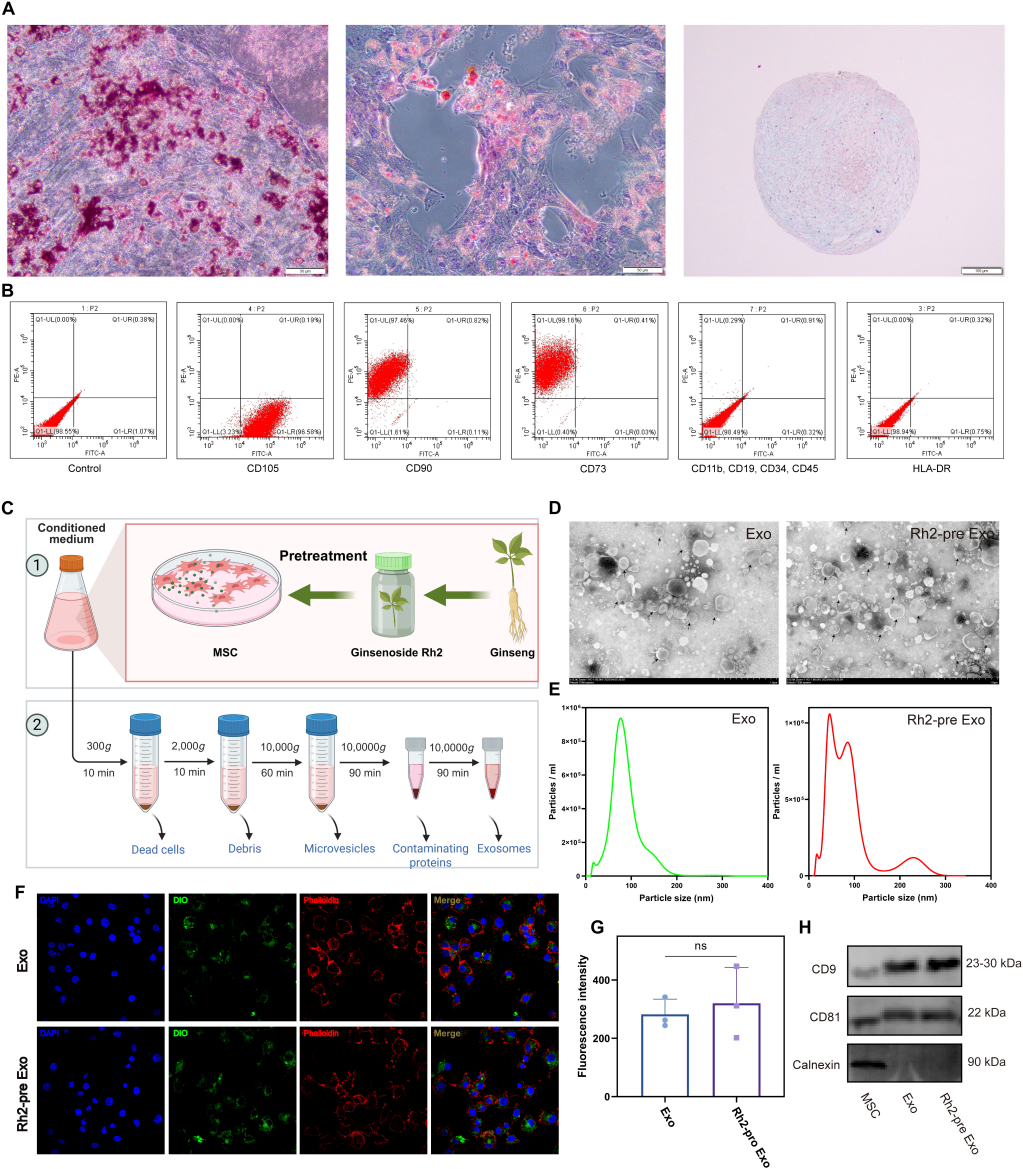
Characterization of MSC and Rh2-pre Exo. (A) Representative images of MSC osteogenic differentiation, lipogenic differentiation, and chondrogenic differentiation. Scale bar, 50 μm. (B) MSC surface markers identified by flow cytometry. (C) Rh2-pre Exo extraction diagram. (D) Representative TEM images of MSC-exo and Rh2-pre Exo. The black arrows indicate typical exosome structures. Scale bar, 1 μm. (E) NTA results of MSC-exo and Rh2-pre Exo. (F) Experimental results of exosome uptake by RAW264.7 cells with LPS stimulation. The green fluorescence represents exosomes stained with DIO, the orange-red fluorescence represents the cytoskeleton stained with SF555-labeled phalloidin, and the blue fluorescence represents the cell nuclei stained with DAPI. Scale bar, 50 μm. (G) Results of statistical analysis of fluorescence intensity of exosome uptake. (H) Results of WB identification of exosome marker proteins.

To assess the differentiation potential of the MSCs used, we conducted experiments targeting osteogenic, adipogenic, and chondrogenic differentiation. The findings demonstrated that MSCs successfully differentiated into calcium nodules, lipid droplets, and chondrospheres upon induction with specific conditioned media (Fig. 1A, from left to right). Moreover, flow cytometry analysis revealed that MSCs were positive for CD90, CD105, and CD73 while showing negative expression for CD11b, CD19, CD34, CD45, and HLA-DR (Fig. 1B).TEM revealed that both MSC-exo and Rh2-pre Exo exhibited a characteristic bilayer phospholipid membrane structure (Fig. 1D), with most exosomes ranging in size from 50 to 180 nm (Fig. 1E). To evaluate the stability and purity of MSC-derived exosomes following Rh2 treatment, we measured the protein concentration of the 2 purified exosome groups using a BCA assay (Fig. S1A) and quantified their size distribution based on fluorescence intensity analysis (Fig. S1B). The results showed no significant differences in total protein content or particle size between Rh2-pretreated exosomes and untreated exosomes, indicating that 24-h Rh2 stimulation does not affect the overall stability or purity of exosomes secreted by MSCs (Fig. S1). Exosome uptake assays demonstrated efficient internalization of both MSC-exo and Rh2-pre Exo by LPS-stimulated RAW264.7 cells (Fig. 1F), with no statistically significant difference in the uptake levels between the 2 groups (Fig. 1G). LPS-unstimulated RAW264.7 cells exhibit minimal uptake of MSC-exo or Rh2-pre Exo (Fig. S2). These findings suggest that MSC-exo and Rh2-pre Exo exhibit strong targeting efficiency toward inflammatory macrophages. Additionally, WB analysis confirmed the presence of exosomal markers CD9 and CD81 in both MSC-exo and Rh2-pre Exo, while the endoplasmic reticulum marker calnexin was absent (Fig. 1H).

### Rh2-pre Exo inhibits proliferation and migration of inflammatory macrophages

**Fig. 2.**
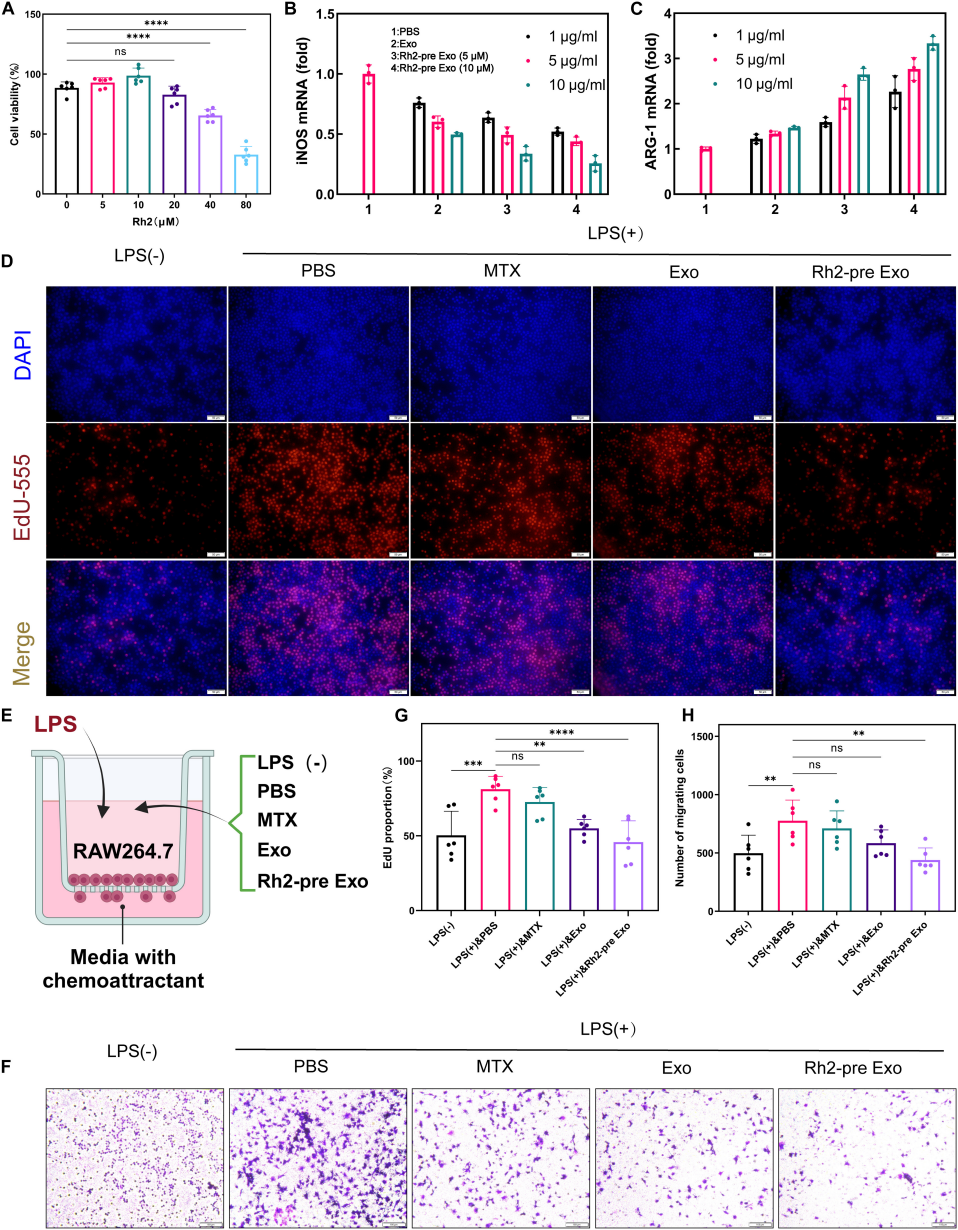
Rh2-pre Exo inhibits proliferation and migration of inflammatory macrophages. (A) Results of experimental determination of the optimum concentration of CCK-8 for Rh2-pretreated MSC. (B) RT-qPCR experiments were performed to detect the expression of INOS under different treatment conditions. (C) RT-qPCR experiments were performed to detect the expression of ARG1 under different treatment conditions. (D) EdU proliferation assay results. RAW264.7 macrophages were treated with extracellular vesicles (EVs) (10 μg/ml) for 24 h. Scale bar, 50 μm. (E) Schematic of the Transwell assay. (F) Transwell migration assay results. RAW264.7 macrophages were treated with EVs (10 μg/ml) for 24 h. Scale bar, 100 μm. (G) Bar chart showing the statistical analysis of EdU-positive cell proportions across different groups. *N* = 6. (H) Bar chart depicting the statistical analysis of migrating cell counts in various groups. *N* = 6. *P < 0.05, **P < 0.01, ***P < 0.005, ****P < 0.001.

First, we utilized the CCK-8 assay to determine the optimal concentration of Rh2 for treating MSCs. As shown in Fig. 2A, concentrations of 5 and 10 μM Rh2 did not significantly affect the cell viability of MSCs. To further establish the optimal concentration of Rh2, we conducted RT-qPCR to assess the impact of 5 and 10 μM Rh2 on macrophage phenotypes, with INOS serving as a marker for M1 macrophages and ARG1 for M2 macrophages. The results, depicted in Fig. 2B and C, indicate that MSCs pretreated with 10 μM Rh2 secreted exosomes that exhibited a stronger ability to suppress the inflammatory phenotype of macrophages. Additionally, the inhibitory effects of Rh2-pro Exo and Exo on the inflammatory phenotype of macrophages were found to be dose-dependent. Specifically, 10 μg/ml of Rh2-pre Exo or Exo demonstrated a more pronounced capacity to inhibit the inflammatory phenotype of macrophages. Therefore, in subsequent experiments, we selected exosomes secreted by MSCs pretreated with 10 μM Rh2, with both Rh2-pre Exo and Exo administered at a concentration of 10 μg/ml. In Fig. 2D, we employed the EdU assay to verify the ability of Rh2-pre Exo to inhibit the proliferation of inflammatory macrophages, and the results showed that Rh2-pre Exo significantly suppressed the proliferation of these cells. Furthermore, the inhibitory effect of Rh2-pre Exo on inflammatory macrophage proliferation was stronger than that of Exo and other drugs (Fig. 2G). Figure 2E presents a schematic of the Transwell assay. The results of the Transwell assay, shown in Fig. 2F, indicate that Rh2-pre Exo significantly inhibited the migration of inflammatory macrophages, and this inhibitory effect was also stronger than that of Exo and other drugs (Fig. 2H).

### Rh2-pre Exo inhibits macrophage inflammatory phenotype and up-regulates anti-inflammatory phenotype

**Fig. 3.**
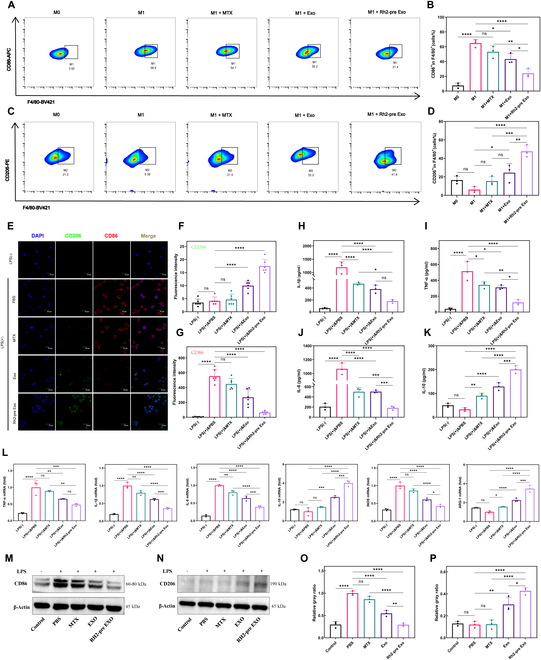
Rh2-pre Exo inhibits macrophage inflammatory phenotype and up-regulates anti-inflammatory phenotype. We treated RAW264.7 macrophages in each group with 10 μg/ml of EVs for 24 h, after which cells or culture supernatants were immediately collected for subsequent analyses. (A) Flow cytometry results of CD86 expression. (B) Bar chart of CD86 expression analyzed by flow cytometry. *N* = 3. (C) Flow cytometry results of CD206 expression. (D) Bar chart of CD206 expression analyzed by flow cytometry. *N* = 3. (E) Results of immunofluorescence staining for CD206 and CD86. Scale bar, 30 μm. (F) The bar chart displays the statistical results of the fluorescence intensity of CD206 in different groups. *N* = 6. (G) The bar chart displays the statistical results of the fluorescence intensity of CD86 in different groups. *N* = 6. (H) ELISA was used to measure IL-1β levels in the supernatant of THP-1 cells from different treatment groups. *N* = 3. (I to K) ELISA was employed to assess the levels of IL-6, TNF-α, and IL-10 in the supernatant of RAW264.7 cells across various treatment groups. *N* = 3. (L) The expression levels of TNF-α, IL-1β, IL-6, IL-10, INOS, and ARG1 in RAW264.7 cells were analyzed using RT-qPCR. *N* = 3. (M and N) Western blot (WB) analysis was conducted to examine the protein expression levels of CD86 and CD206 in RAW264.7 cells. *N* = 3. (O) Bar graphs were generated to quantify the grayscale analysis of CD86 protein expression. (P) Bar graphs were generated to quantify the grayscale analysis of CD206 protein expression. *N* = 3. *P < 0.05, **P < 0.01, ***P < 0.001, ****P < 0.0001.

To investigate the effects of Rh2-pre Exo on macrophage phenotypes, we first conducted flow cytometry experiments. CD86 was used as a marker for M1 macrophages, and CD206 as a marker for M2 macrophages. As shown in Fig. 3A and B, the proportion of CD86^+^ macrophages significantly decreased in the Rh2-pre Exo group, with a more pronounced effect compared to the Exo group and other treatment groups. Furthermore, Fig. 3C and D shows that the proportion of CD206^+^ macrophages significantly increased in the Rh2-pre Exo group, again surpassing the effects observed in the Exo group and other treatments. Immunofluorescence analysis further confirmed these findings, with Rh2-pre Exo-treated macrophages exhibiting a significant decrease in CD86 protein expression and a marked increase in CD206 expression, with superior efficacy compared to the Exo group and other treatments (Fig. 3E to G).

Additionally, we measured the levels of cytokines in the supernatants from different treatment groups. As illustrated in Fig. 3H to K, Rh2-pre Exo significantly suppressed the expression of pro-inflammatory cytokines IL-1β, IL-6, and TNF-α while promoting the expression of the anti-inflammatory cytokine IL-10. To further validate these results, we performed RT-qPCR and WB analyses, which corroborated the trends in key indicators (Fig. 3L to P). Collectively, these results indicate that Rh2-pre Exo effectively suppresses the inflammatory phenotype of macrophages and promotes an anti-inflammatory phenotype. Additionally, we used THP-1 cells to assess the ability of Rh2-pre Exo to induce the differentiation of monocytes into M2 macrophages. Flow cytometry results showed that Rh2-pre Exo significantly promoted the expression of CD206 (Fig. S3A and B). Immunofluorescence staining further confirmed the enhanced expression of CD206 (Fig. S3C and D). WB analysis demonstrated a marked up-regulation of Arg1 expression following Rh2-pre Exo treatment (Fig. S3E and F). Together, these results indicate that Rh2-pre Exo can effectively induce the polarization of monocytes toward the M2 macrophage phenotype.

### Rh2-pre Exo ameliorates arthritis symptoms in CIA rats

**Fig. 4.**
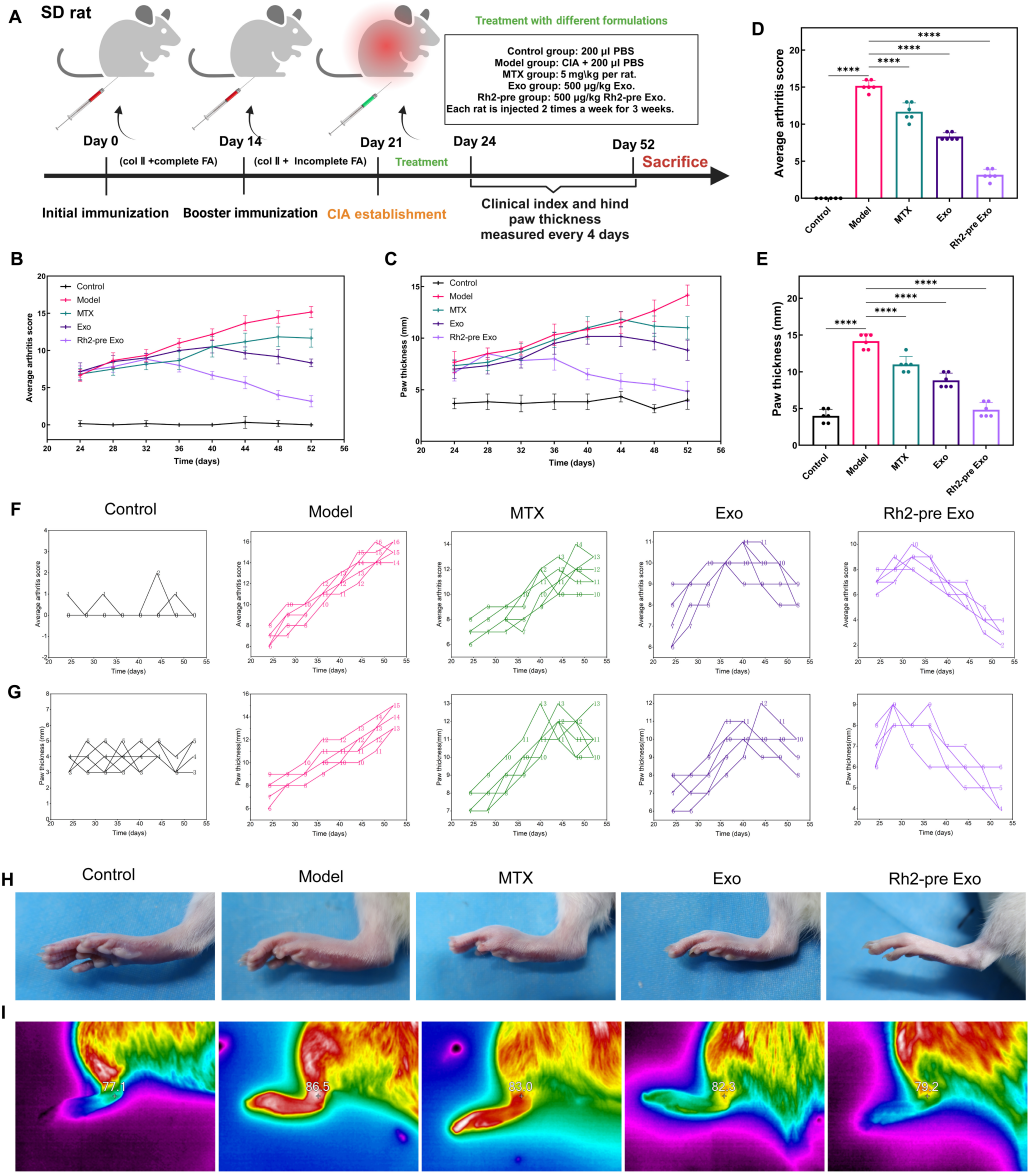
Rh2-pre Exo ameliorates arthritis symptoms in CIA rats. (A) Construction and treatment protocol of CIA rat model. (B) Results of average arthritis scores in different treatment groups. Arthritis scores were recorded every 4 d until the end of the experiment. (C) Results of paw thickness in different treatment groups. Paw pad thickness was also measured every 4 d throughout the duration of the study. (D) Statistical results of average arthritis scores on day 52 in each group. *N* = 6. (E) Statistical results of paw thickness on day 52 in each group. *N* = 6. (F) Changes in the average arthritis score of individual rats. (G) Changes in the paw thickness of individual rats. (H) Typical photographs of rat paws. (I) Representative infrared (IR) images of rat paw. Use the RGB color mode. ****P < 0.0001.

To evaluate the therapeutic effects of Rh2-pre Exo on RA, we constructed a CIA model in rats and assigned them to different treatment groups (Fig. 4A). The average arthritis scores and paw thickness were used to assess the severity of arthritis. As shown in Fig. 4B, the arthritis scores in the model group exhibited a continuous upward trend throughout the observation period. Although the MTX treatment group also showed an increasing trend in arthritis scores, the extent of increase was lower than that of the model group. In contrast to both the model and MTX groups, the arthritis scores in the Exo group began to decline on day 40, while the Rh2-pre Exo group showed a downward trend as early as day 32. Additionally, as illustrated in Fig. 4C, the paw thickness of the model group displayed a persistent increase during the observation period, with the MTX treatment group ceasing to increase on day 44. Unlike the model and MTX groups, the Exo group began to show a reduction in paw thickness on day 40, and the Rh2-pre Exo group exhibited a decreasing trend as early as day 28. On day 52, the arthritis scores of the Rh2-pre Exo group were significantly lower than those of the other groups (Fig. 4D), and on the same day, the paw thickness in the Rh2-pre Exo group was also significantly reduced compared to the other groups (Fig. 4E). Figure 4F and G illustrates the changes in arthritis scores and paw thickness across all groups. Figure 4H presents typical photographs of the rats’ paws, clearly showing significant redness and swelling in the model group, while the Rh2-pre Exo group exhibited the least swelling. Lastly, we utilized an infrared thermography device to measure the paw temperatures of the rats in each group. The results indicated that the paw temperature in the Rh2-pre Exo group was significantly lower than that in the other groups (Fig. 4I).

### Rh2-pre Exo ameliorates arthritis symptoms in CIA mice

**Fig. 5.**
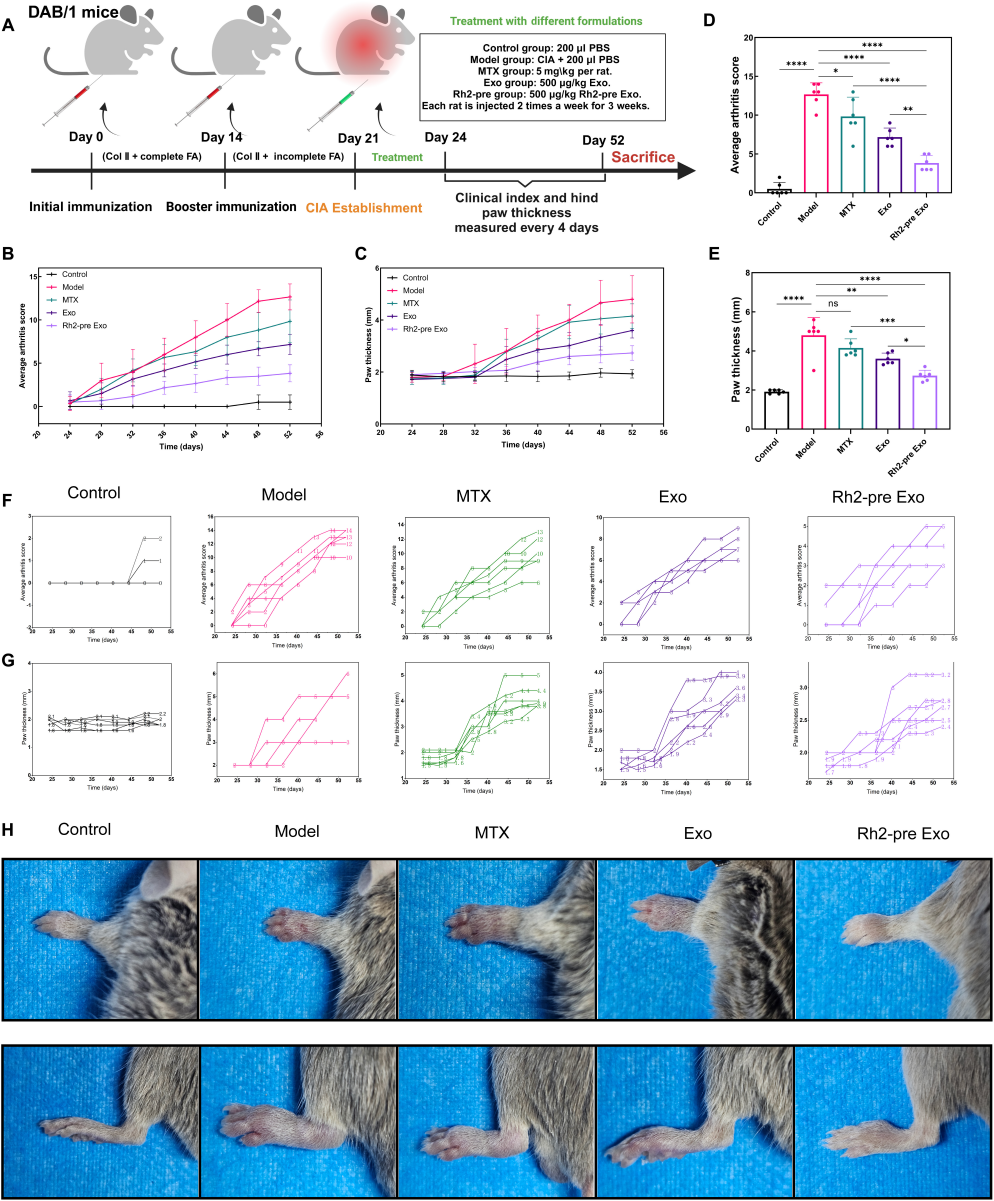
Rh2-pre Exo improves arthritis symptoms in CIA mice. (A) Construction and treatment protocol of CIA rat model. (B) Results of average arthritis scores in different treatment groups. Arthritis scores were recorded every 4 d until the end of the experiment. (C) Results of paw thickness in different treatment groups. Paw pad thickness was also measured every 4 d throughout the duration of the study. (D) Statistical results of average arthritis scores on day 52 in each group. *N* = 6. (E) Statistical results of paw thickness on day 52 in each group. *N* = 6. (F) Changes in the average arthritis score of individual rats. (G) Changes in the paw thickness of individual rats. (H) Typical photographs of rat paws. *P < 0.05, **P < 0.01, ***P < 0.001, ****P < 0.0001.

To further evaluate the therapeutic effects of Rh2-pre Exo on RA, we also established a CIA mouse model and assigned them to different treatment groups (Fig. 5A). The average arthritis scores and paw thickness were similarly used to assess the severity of arthritis. Unlike the CIA rat model, we observed that during the observation period, all groups except the control group showed a continuous upward trend in arthritis scores; however, the increase in the Rh2-pre Exo group was less pronounced compared to the other groups (Fig. 5B). Additionally, as shown in Fig. 5C, paw thickness also exhibited a continuous upward trend in all groups except the control group, with the Rh2-pre Exo group showing a lesser increase compared to the other groups. On day 52, the arthritis scores in the Rh2-pre Exo group were significantly lower than those in the other groups (Fig. 5D). Similarly, on day 52, the paw thickness in the Rh2-pre Exo group was also significantly reduced compared to the other groups (Fig. 5E). Figure 5F and G shows the changes in arthritis scores and paw thickness across all groups. Figure 5H presents representative photographs of the paws in each group, where evident swelling and redness were observed in the model group, while the Rh2-pre Exo group exhibited the least swelling and redness.

**Fig. 6.**
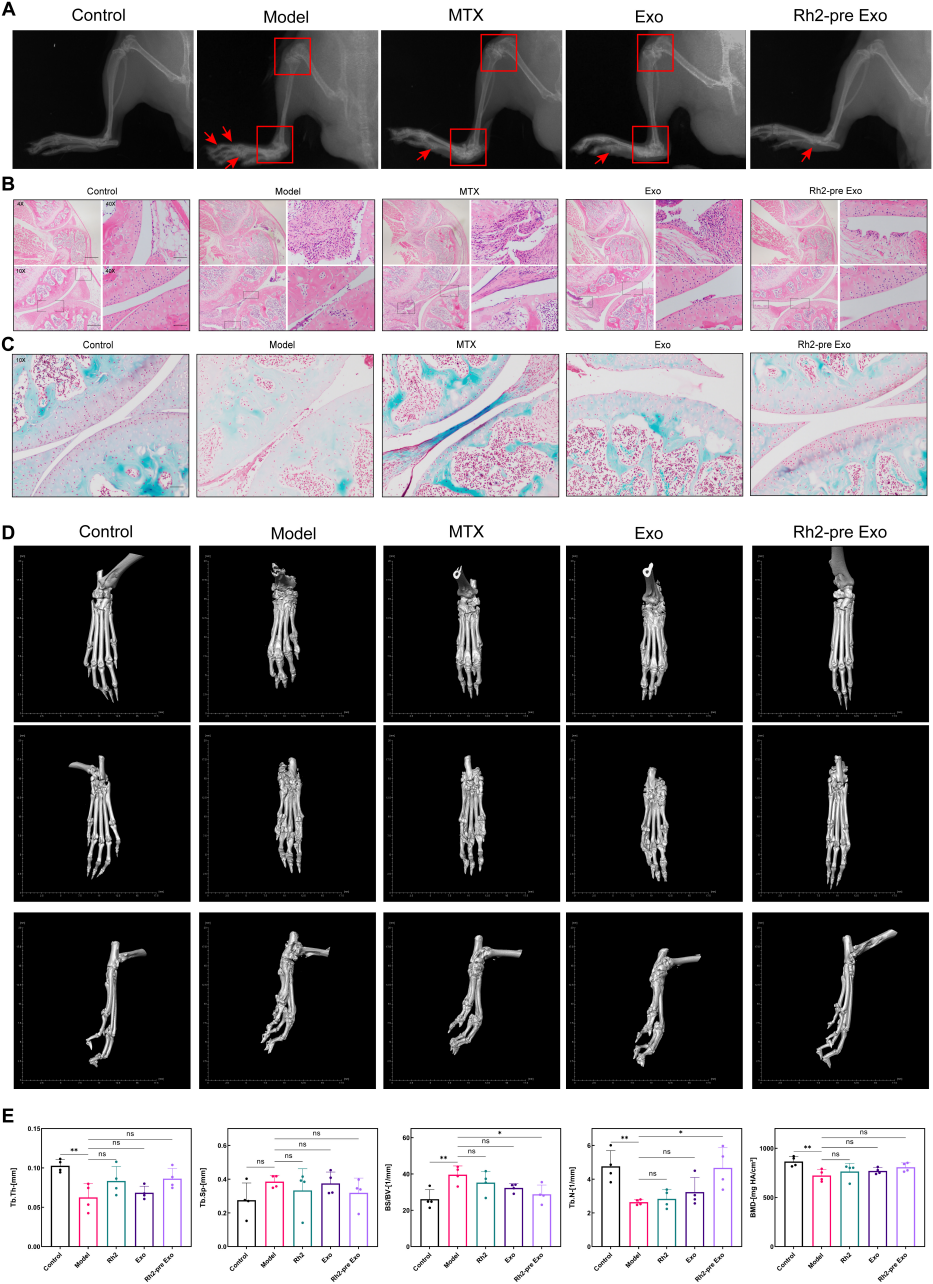
Results of x-ray, H&E staining, Safranin O/Fast Green staining, and micro-CT examination of specimens. (A) Results of x-ray imaging of mouse joints. (B) H&E staining results of mouse knee joints. (C) Safranin O/Fast Green staining results of mouse knee joints. Scale bar (4×), 500 μm; scale bar (10×), 200 μm; scale bar (20×), 100 μm; scale bar (40×), 50 μm. (D) Micro-CT 3D reconstruction results of mouse paws. (E) Results of the statistical analysis for bone mineral density (BMD), trabecular thickness (Tb.Th), trabecular separation (Tb.Sp), trabecular number (Tb.N), and the ratio of bone surface area to bone volume (BS/BV). *N* = 4. *P < 0.05, **P < 0.01.

To further evaluate the therapeutic effects of Rh2-pre Exo on arthritis in mice, we first performed x-ray imaging of the hind limbs, focusing on the knee joints, ankle joints, and paws. As shown in Fig. 6A, the model group exhibited disorganized joint structures, severe osteophyte formation, joint deformities, and dislocated small joints in the paws. In contrast, the Rh2-pre Exo treatment group displayed generally normal joint structures, with only minor osteophyte formation in the paws. The MTX and Exo groups showed partial relief from arthritis, but joint structure damage and severe osteophyte formation were still evident.

Next, we performed H&E staining and Safranin O/Fast Green staining on the pathological sections of the knee and ankle joints. H&E staining was used to assess joint structure and synovial hyperplasia. As shown in Fig. 6B, the control group had normal joint spaces, smooth joint surfaces, and no synovial hyperplasia. In contrast, the model group had a complete loss of joint space, rough joint surfaces, and severe synovial hyperplasia. Compared to the model group, the Rh2-pre Exo treatment group displayed restored joint spaces, smooth joint surfaces, and only mild synovial hyperplasia. The MTX and Exo groups exhibited varying degrees of improvement, but not to the extent seen in the Rh2-pre Exo group.

Safranin O/Fast Green staining was used to assess cartilage integrity in the knee joints. As shown in Fig. 6C, the cartilage in the knee joints of the model group was completely worn down, while the MTX group also showed severe cartilage loss. The Exo group had partial cartilage repair, but the joint surfaces remained uneven. In contrast, the Rh2-pre Exo group showed relatively intact cartilage. We also performed H&E and Safranin O/Fast Green staining on the ankle joints, with results similar to those seen in the knee joints (Fig. S4).

To assess the impact of different treatments on bone quality, we performed micro-CT imaging of the mouse paws. As shown in Fig. 6D, the model group exhibited significant osteophyte formation, disorganized small joint structures, and severe bone erosion. The Rh2-pre Exo treatment group showed marked alleviation of bone erosion and clear joint structures. The MTX and Exo groups displayed varying degrees of improvement in bone erosion, but their effects were not as pronounced as those in the Rh2-pre Exo group.

As shown in Fig. S5, we selected a fixed region at the distal tibia for 3-dimensional reconstruction and measured parameters reflecting bone quality, including bone mineral density (BMD), trabecular thickness (Tb.Th), trabecular number (Tb.N), trabecular separation (Tb.Sp), and bone surface area to bone volume ratio (BS/BV), followed by statistical analysis. As shown in Fig. 6E, the model group exhibited decreased BMD, Tb.N, and Tb.Th values, along with increased Tb.Sp and BS/BV values, indicating severe bone loss. Although the MTX and Exo groups showed slight improvement in bone quality in the 3D reconstruction, the differences in the measured parameters were not statistically significant. In contrast, the Rh2-pre Exo group showed a significant increase in Tb.N and a marked decrease in BS/BV compared to the model group.

### Rh2-pre Exo exerts anti-inflammatory effects and regulates macrophage polarization by targeting inflamed joints

**Fig. 7.**
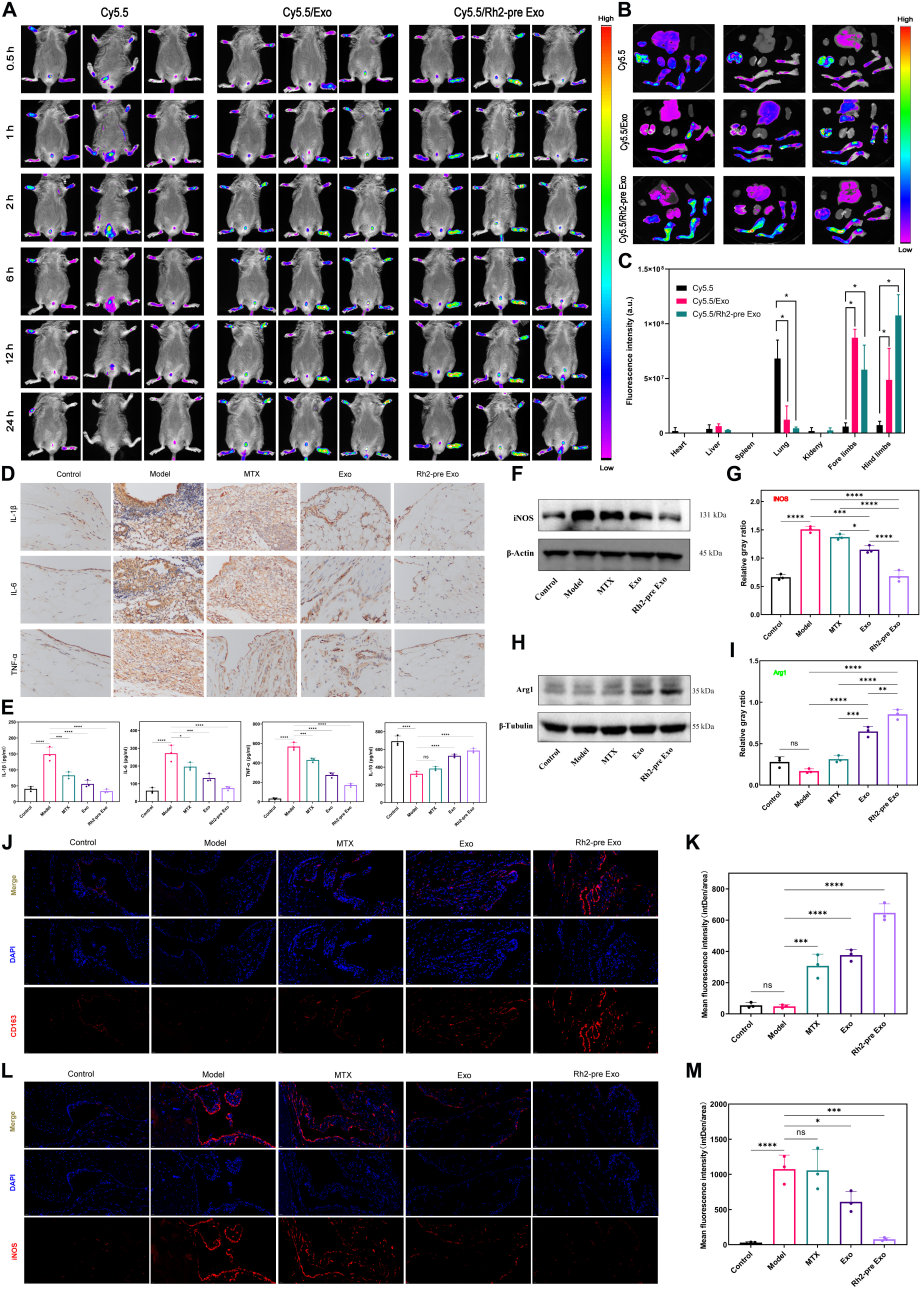
Rh2-pre Exo exerts anti-inflammatory effects and regulates macrophage polarization by targeting inflamed joints. *(*A) In vivo imaging results in small animals: Cy5.5-labeled Rh2-pre Exo and Cy5.5-labeled Exo. MSC-exo group: 500 μg/kg of Cy5.5-labeled MSC-exo. Rh2-pre Exo group: 500 μg/kg of Cy5.5-labeled Rh2-pre Exo. Control group: 500 μl of Cy5.5 solution. (B) Fluorescence distribution across organs and limbs. (C) Statistical analysis of fluorescence intensity in organs and limbs. *N* = 3. (D) Immunohistochemistry results for IL-1β, IL-6, and TNF-α in the synovial tissues of mice from different treatment groups. Scale bar, 50 μm. (E) ELISA results for IL-1β, IL-6, TNF-α, and IL-10 in the synovial tissue homogenates of mice from different treatment groups. *N* = 3. (F) WB analysis of INOS protein in the synovial tissues of mice from different treatment groups. (G) Grayscale statistical analysis of INOS protein expression in synovial tissues from different treatment groups. *N* = 3. (H) WB analysis of Arg1 protein in the synovial tissues of mice from different treatment groups. (I) Grayscale statistical analysis of Arg1 protein expression in synovial tissues from different treatment groups. *N* = 3. (J) Immunofluorescence analysis of CD163 protein in the synovial tissues of mice from different treatment groups. Scale bar, 20 μm. (K) Statistical analysis of average fluorescence intensity for CD163 protein in the synovial tissues of mice from different treatment groups. *N* = 3. (L) Immunofluorescence analysis of INOS protein in the synovial tissues of mice from different treatment groups. Scale bar, 20 μm. (M) Statistical analysis of average fluorescence intensity for INOS protein in the synovial tissues of mice from different treatment groups. *N* = 3. *P < 0.05, **P < 0.01, ***P < 0.001, ****P < 0.0001.

Exosome targeting to inflamed joints is a prerequisite for its therapeutic effects. To verify the in vivo targeting ability of Rh2-pre Exo, we conducted small animal live imaging experiments. Rh2-pre Exo was labeled with Cy5.5 and injected into mice via the tail vein. Cy5.5-labeled Exo and Cy5.5 alone were used as controls. As shown in Fig. 7A, the Cy5.5 group exhibited some fluorescence in the paws, but the intensity began to fade after 2 h and almost disappeared by 24 h. In contrast, the paws of the Cy5.5-labeled Rh2-pre Exo and Cy5.5-labeled Exo groups showed significantly higher fluorescence intensity. The fluorescence in the Rh2-pre Exo and Exo groups peaked around 6 h and persisted until 24 h. After 24 h, mice were dissected to observe exosome accumulation in various organs. As shown in Fig. 7B and C, fluorescence in the Cy5.5-labeled Rh2-pre Exo and Cy5.5-labeled Exo groups was primarily concentrated in the limbs, with a small amount retained in the liver and lungs. In contrast, the Cy5.5 group exhibited weaker limb fluorescence and stronger fluorescence in the lungs. These results indicate that Rh2-pre Exo possesses targeted ability to inflamed regions.

Next, we performed immunohistochemistry to assess the expression of inflammatory cytokines IL-1β, IL-6, and TNF-α in the mouse joints. As shown in Fig. 7D, the model group displayed severe synovial hyperplasia with significantly increased expression of IL-1β, IL-6, and TNF-α. In the Rh2-pre Exo group, the synovium appeared normal, and the expression levels of IL-1β, IL-6, and TNF-α were markedly reduced. The MTX and Exo groups had lower cytokine expression compared to the model group but higher levels than the Rh2-pre Exo group. We further confirmed these findings through ELISA, measuring the levels of IL-1β, IL-6, TNF-α, and IL-10 in synovial tissue homogenates, with results consistent with the immunohistochemistry data (Fig. 7E).

WB analysis was also performed to detect the expression of INOS and Arg1 proteins in the synovial tissue. As shown in Fig. 7F to I, the model group exhibited significantly elevated INOS expression, which was notably reduced after Rh2-pre Exo treatment. Meanwhile, Arg1 expression was low in the model group but significantly increased following Rh2-pre Exo treatment. The effects of MTX and Exo treatments were less pronounced than those of Rh2-pre Exo. These findings suggest that Rh2-pre Exo exerts anti-inflammatory effects by modulating macrophage polarization phenotypes.

Lastly, immunofluorescence staining of the synovial tissue was used to further validate these conclusions, as shown in Fig. 7J to M.

### Proteomic identification of altered proteins and potentially affected pathways in the synovial tissue of CIA mice following Rh2-pre Exo treatment

**Fig. 8.**
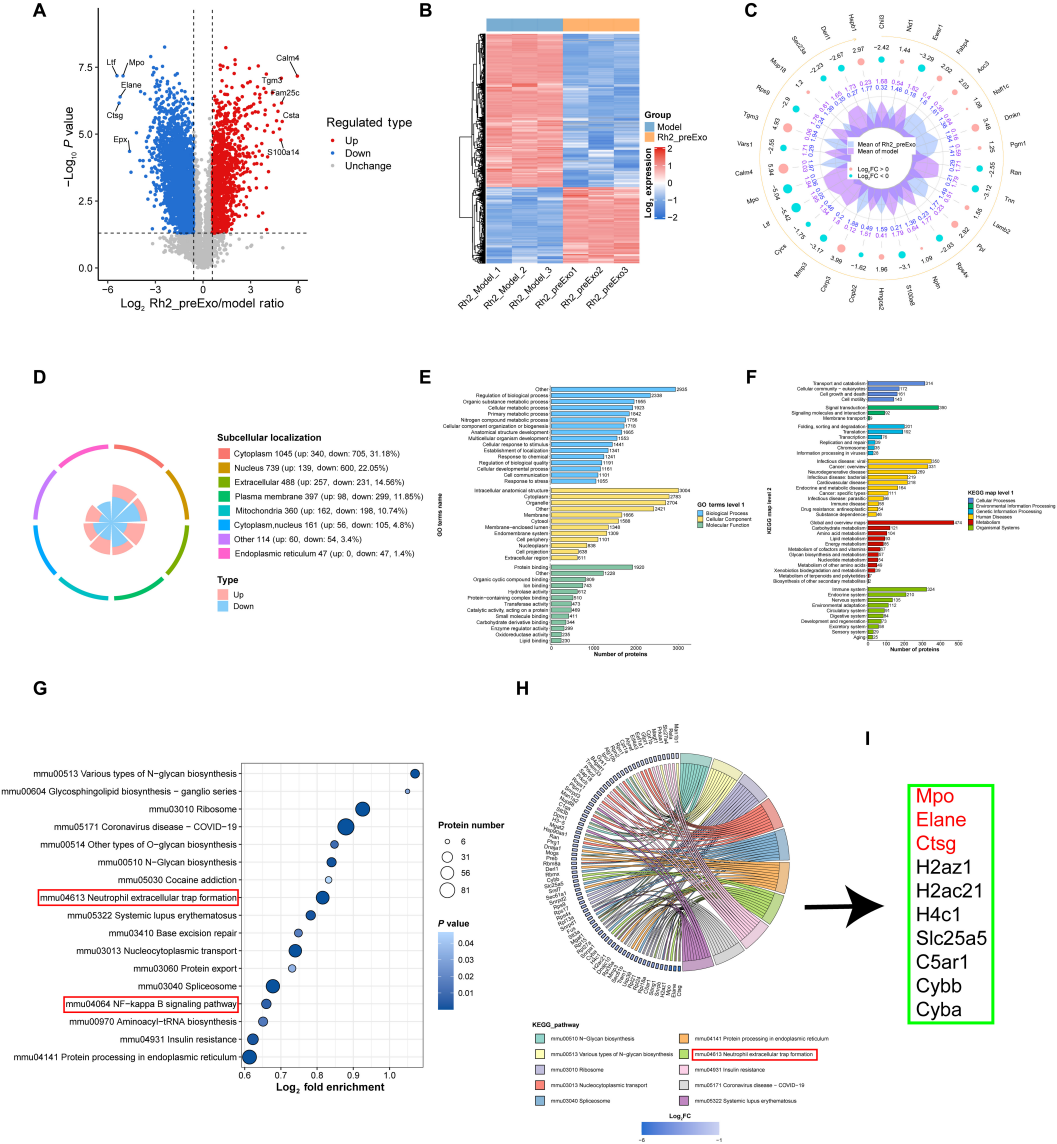
Proteomic identification of altered proteins and potentially affected pathways in the synovial tissue of CIA mice following Rh2-pre Exo treatment. (A) Volcano plot of differential proteins. (B) Heatmap of differential proteins. (C) Radar plot of differential proteins. (D) Subcellular localization classification of differential proteins. (E) GO secondary classification of differential proteins. (F) KEGG functional classification of differential proteins. (G) Bubble plot for KEGG pathway enrichment analysis of differential proteins. (H) Chord diagram for functional enrichment of differential proteins. (I) Differential proteins enriched in the NET formation pathway in the chord diagram.

We utilized proteomics sequencing to compare the changes in synovial tissue proteins between the CIA mouse model group and the Rh2-pre Exo treatment group. Figure S6 shows that, following Rh2-pre Exo treatment, 1,112 proteins were up-regulated, while 2,241 proteins were down-regulated. Figure 8A presents the results of the differential protein volcano plot, where red dots represent significantly up-regulated proteins, blue dots indicate significantly down-regulated proteins, and gray dots show no significant difference. The top 5 up-regulated and down-regulated proteins (based on the absolute value of log_2_ ratio) are labeled in the figure.

Figure 8B displays the heatmap of differential proteins, illustrating the relative expression levels of multiple differential proteins across different samples and showing their clustering relationships. Each row represents a differential protein, and each column corresponds to a sample. Red indicates high expression, blue represents low expression, and gray signifies undetectable quantities in the respective sample. A radar plot of the top 30 differential proteins is provided to show their relative expression levels. The first ring of the radar plot represents the differential proteins; the second ring displays log_2_-transformed ratio values, with pink indicating up-regulation and light blue indicating down-regulation. Larger dots represent greater fold changes. The third ring reflects the average quantitative levels between the 2 groups, where sharp peaks indicate highly expressed proteins (Fig. 8C).

These results collectively demonstrate significant alterations in the synovial proteins of CIA mice following Rh2-pre Exo treatment. Figure 8D depicts the number of up- and down-regulated proteins within various subcellular structures, with cytoplasmic proteins comprising the largest proportion (31.18%).

Figure 8E shows the results of the GO secondary classification of differential proteins. The *x* axis indicates the number of proteins within each category, while the *y* axis represents the secondary functional classifications within GO’s primary categories (Biological Process, Cellular Component, and Molecular Function). The results indicate that differential proteins are enriched in the secondary functional categories of regulation of biological processes, intracellular anatomical structures, and protein binding.

Figure 8F presents the KEGG pathway classification analysis of differential proteins, with the *x* axis showing the number of proteins in each category and the *y* axis listing the secondary functional classifications within KEGG’s primary categories (Metabolism, Genetic Information Processing, Environmental Information Processing, Cellular Processes, Organismal Systems, Human Diseases, and Drug Development). The results suggest that differential proteins are enriched in Signal Transduction, Immune Disease, and Immune System pathways, indicating that Rh2-pre Exo treatment may affect intracellular processes such as immune system signaling and protein binding.

We further performed KEGG pathway enrichment analysis on the differentially expressed proteins in the comparison groups, with a bubble chart (Fig. 8G) showing the top 20 most significantly enriched pathways. Among them, the enrichment of neutrophil extracellular trap (NET) formation and the NF-κB signaling pathway caught our attention. Figure 8H illustrates the enriched pathways via a chord diagram, and Fig. 8I highlights the significantly altered proteins within the NET formation pathway. These findings suggest that Rh2-pre Exo may exert its therapeutic effects on arthritis by modulating NET formation and the NF-κB signaling pathway.

### Proteomic result validation

**Fig. 9.**
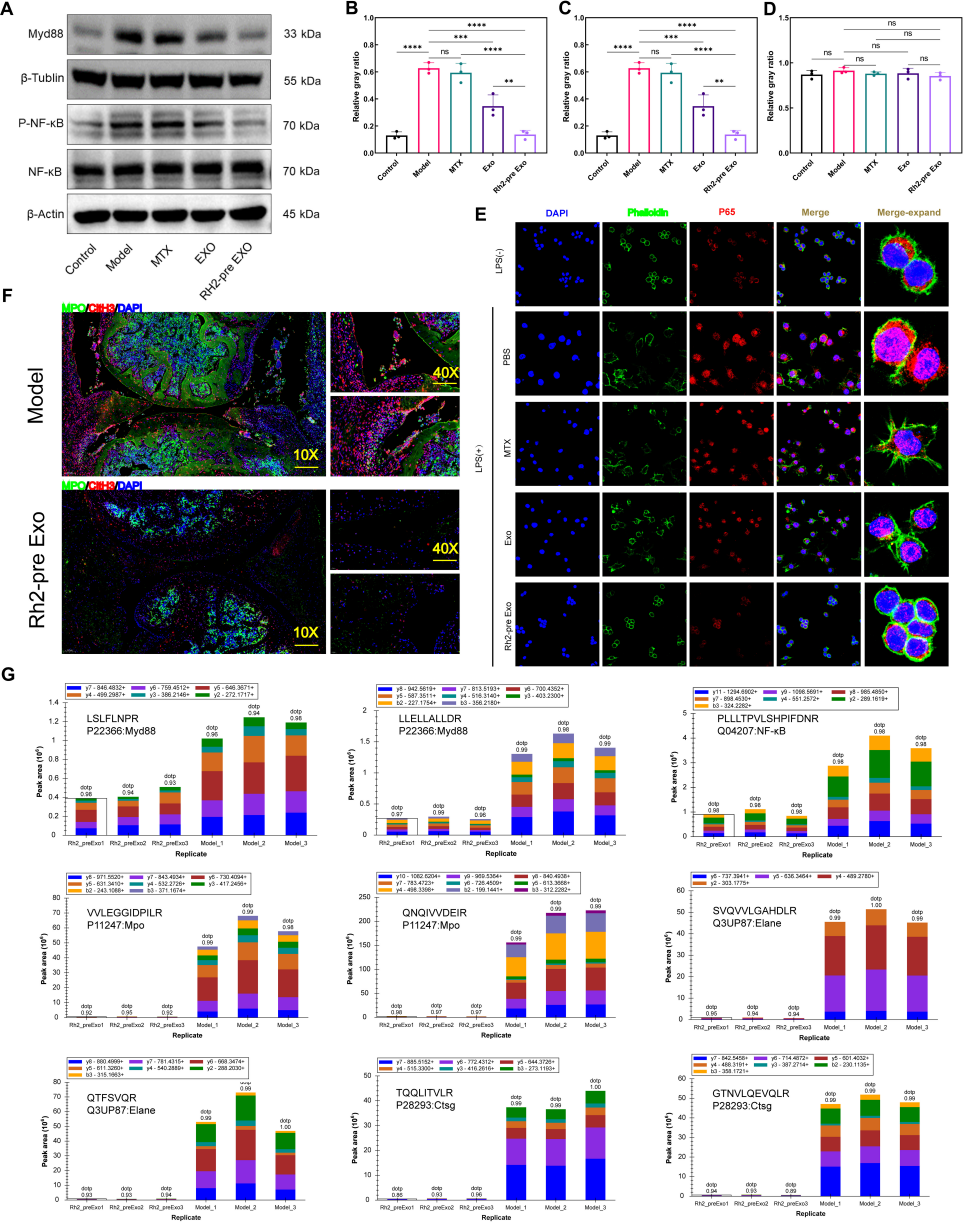
Proteomic results validation. (A) Expression levels of Myd88, P-NF-κB, and NF-κB across different treatment groups. (B) Statistical analysis of the grayscale values for Myd88 protein expression. *N* = 3. (C) Statistical analysis of the grayscale values for P-NF-κB protein expression. *N* = 3. (D) Statistical analysis of the grayscale values for NF-κB protein expression. *N* = 3. (E) Immunofluorescence results showing nuclear translocation of NF-κB P65 in macrophages. Green represents the cytoskeleton labeled with phalloidin, blue indicates cell nuclei stained with DAPI, and red represents P65. Scale bar, 50 μm. (F) Immunofluorescence results in mouse joints. Green indicates MPO, red represents CitH3, and blue shows cell nuclei labeled with DAPI. Scale bar (10x), 200 μm; scale bar (40x), 50 μm. (G) Quantitative results of target proteins using MS-based PRM. The quantification is based on peak area measurements. The figure displays the distribution of fragment ion peak areas for selected peptides across 6 samples. Each protein was quantified using 2 unique peptides; however, some proteins were only identified with one peptide due to sensitivity limitations. *P < 0.05, **P < 0.01, ***P < 0.001, ****P < 0.0001.

In the aforementioned proteomic sequencing experiment, we identified that Rh2-pre Exo may exert its therapeutic effects on CIA mice by influencing the NF-κB pathway and NET formation. In this section, we validate the proteomic findings through relevant experiments. As shown in Fig. 9A, we first assessed the expression levels of key NF-κB pathway proteins in the synovial tissue of mice using WB analysis. The results indicate that the expression levels of Myd88 and phosphorylated NF-κB (P-NF-κB) proteins in the Rh2-pre Exo group were significantly reduced compared to the model group. Additionally, the Exo group also exhibited a decrease in Myd88 and P-NF-κB expression, although the reduction was less pronounced than that observed in the Rh2-pre Exo group (Fig. 9B to D).

Moreover, we evaluated the nuclear translocation of NF-κB P65 in different treatment groups using immunofluorescence assays. The results, presented in Fig. 9E, show that the nuclear levels of NF-κB P65 protein in the Rh2-pre Exo group were significantly decreased compared to the model group. The Exo group also demonstrated a certain degree of reduction in NF-κB P65 nuclear translocation, but again, the decrease was not as significant as that in the Rh2-pre Exo group. These findings suggest that Rh2-pre Exo treatment markedly inhibits the NF-κB pathway and restricts the nuclear translocation of P65 protein in inflammatory macrophages. As illustrated in Fig. 9F, we further confirmed the expression levels of NET formation-related proteins MPO and CitH3 in the joints of mice through immunofluorescence assays. The results indicated a significant reduction in MPO and CitH3 expression levels in the synovial tissue of mice following Rh2-pre Exo treatment.

To validate the proteomic results with greater accuracy, we utilized parallel reaction monitoring (PRM) technology for the quantitative confirmation of the target proteins (Fig. 9G). In this experiment, each protein was quantified based on 2 unique peptides; however, certain proteins were identified with only one peptide due to limitations in sensitivity. The results demonstrated that the fragment ion peak areas of the 2 specific peptides for Myd88 (P22366) (LSLFLNPR, LLELLALLDR) were significantly reduced in the Rh2-pre Exo treatment group. Similarly, the fragment ion peak area of the specific peptide for NF-κB (Q04207) (PLLLTPVLSHPIFDNR) was also significantly diminished in the Rh2-pre Exo group.

Furthermore, the fragment ion peak areas of the 2 specific peptides for MPO (P11247) (VVLEGGIDPILR, QNQIVVDEIR) were markedly reduced in the Rh2-pre Exo group. The 2 specific peptides for Elane (Q3UP87) (SVQVVLGAHDLR and QTFSVQR) and the 2 specific peptides for Ctsg (P28293) (TQQLITVLR and GTNVLQEVQLR) also showed significant decreases in the Rh2-pre Exo group. Notably, Myd88 (P22366) and NF-κB (Q04207) are key proteins in the NF-κB pathway, while MPO (P11247), Elane (Q3UP87), and Ctsg (P28293) are critical proteins in NET formation.

Collectively, these results substantiate the findings from the proteomic sequencing analysis.

### Combined MeRIP-seq and RNA-seq reveal Rh2-pre Exo regulation of CCRL2 mRNA methylation via ALKBH5

**Fig. 10.**
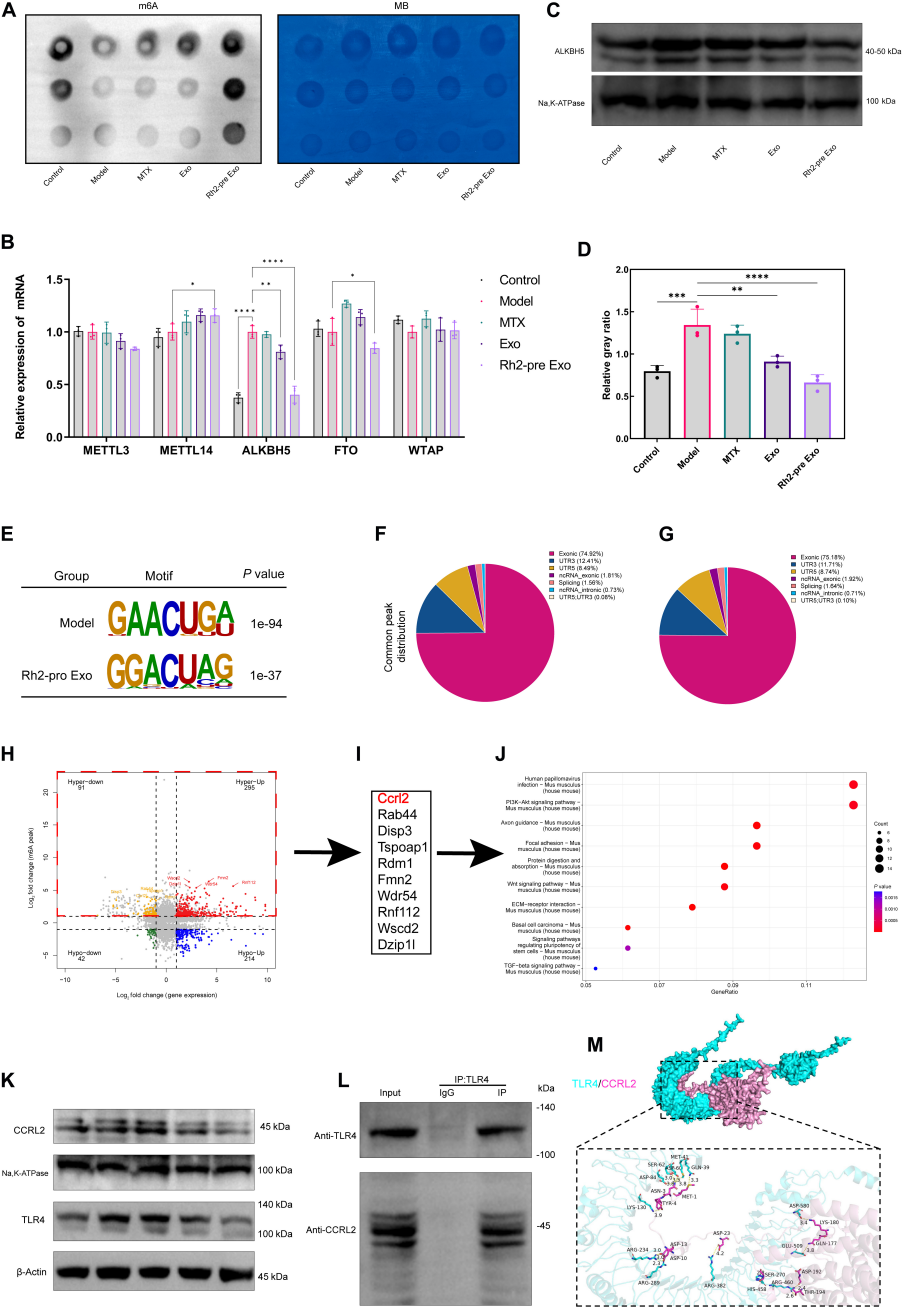
Combined MeRIP-seq and RNA-seq reveal Rh2-pre Exo regulation of CCRL2 mRNA methylation via ALKBH5. (A) M6A levels were assessed across different treatment groups via dot blot assay, with methylene blue staining as a loading control. (B) Expression levels of methylation-related genes were measured by qPCR. *N* = 3. (C) ALKBH5 protein expression was evaluated by WB across different treatment groups. (D) Statistical bar graph showing the grayscale analysis of ALKBH5 protein expression in each treatment group. *N* = 3. (E) Predominant consensus motifs identified in m6A-seq peaks across both groups. (F) Distribution and proportions of m6A peaks across different regions in the model group. (G) Distribution and proportions of m6A peaks across different regions in the Rh2-pre Exo treatment group. (H) Classification of differentially expressed genes (*P* < 0.05; fold change ≥ 2) based on mRNA and m6A methylation levels. (I) Top 10 genes with the most significant differential expression among hypermethylated genes. (J) KEGG pathway enrichment bubble plot showing pathways associated with differentially expressed genes. (K) WB analysis of CCRL2 and TLR4 protein expression across different treatment groups. (L) Co-immunoprecipitation analysis of the interaction between CCRL2 and TLR4 proteins. (M) Molecular docking illustration of the interaction between CCRL2 and TLR4 proteins. *P < 0.05, **P < 0.01, ***P < 0.001, ****P < 0.0001.

To further investigate the molecular mechanism underlying Rh2-pre Exo’s therapeutic effects on RA, we performed MeRIP-seq and RNA-seq analyses on synovial tissue from the model and Rh2-pre Exo-treated groups. First, we used an RNA dot blot assay to measure total m6A levels in the synovial tissue of rats treated with Rh2-pre Exo. As shown in Fig. 10A, m6A levels were significantly higher in the Rh2-pre Exo treatment group compared to the model group. We then used qPCR to assess the expression of methylation-related factors, finding that ALKBH5 exhibited the most pronounced change among these factors, with Rh2-pre Exo treatment significantly down-regulating ALKBH5 expression in the synovial tissue (Fig. 10B). WB further validated the significant reduction of ALKBH5 at the protein level in the Rh2-pre Exo-treated group (Fig. 10C and D). Motif analysis of the model and Rh2-pre Exo groups revealed that the GGACU sequence was highly enriched at m6A sites, consistent with the typical m6A consensus sequence, RRACH (where R = G or A, and H = A, C, or U). The m6A peaks were predominantly located in the coding sequence (CDS) exon regions and the 3’ untranslated regions (UTRs) of mRNA transcripts (Fig. 10E to G). MeRIP-seq analysis showed that synovial tissue from the Rh2-pre Exo group exhibited significant changes in m6A levels in 2,786 genes (*P* < 0.05, m6A peak fold change ≥ 2), with 1,011 genes hypomethylated and 1,775 genes hypermethylated, which were respectively termed hypomethylated and hypermethylated m6A-related genes (Fig. S7). RNA-seq data analysis revealed that 3,104 genes exhibited significant differential expression in the Rh2-pre Exo-treated group (*P* < 0.05, fold change ≥ 2), with 1,008 genes down-regulated and 2,096 genes up-regulated (Fig. S8). Integrating MeRIP-seq and RNA-seq data (Fig. 10H), we identified 295 hypermethylated m6A-related genes that were up-regulated (295 hyper-up), 91 hypermethylated m6A-related genes that were down-regulated (91 hyper-down), 214 hypomethylated m6A-related genes that were up-regulated (214 hypo-up), and 42 hypomethylated m6A-related genes that were down-regulated (42 hypo-down). Given that ALKBH5 mediates m6A demethylation (Fig. 10A to C), these 2,096 hypermethylated genes are predicted to include true ALKBH5 targets. We focused on m6A-hypermethylated transcripts, listing the top 10 genes with the most significant differences (Fig. 10I). We validated these top 10 genes by qPCR and found that CCRL2 had the most significant differential expression, suggesting it as the most likely ALKBH5 target (Fig. S9). We confirmed via WB that Rh2-pre Exo treatment also significantly reduced CCRL2 expression at the protein level in synovial tissue (Fig. 10K). KEGG analysis indicated that several genes were related to focal adhesion (Fig. 10J), which is consistent with the observed reduced inflammatory cell migration and macrophage phenotype shift in the Rh2-pre Exo-treated group.

We attempted to link CCRL2 changes to proteomics results, indicating changes in the NF-κB pathway. TLR4 activation can induce NF-κB pathway activation, typically leading to M1 polarization (pro-inflammatory) in macrophages. WB analysis showed that Rh2-pre Exo significantly reduced TLR4 expression in synovial tissue (Fig. 10K). Furthermore, co-immunoprecipitation confirmed an interaction between CCRL2 and TLR4 (Fig. 10L). Molecular docking was performed to investigate the binding interactions between CCRL2 and TLR4, revealing a potential interaction with a binding energy of −4.2 kcal/mol. Hydrogen bonds are represented by yellow dashed lines, and amino acid residues involved in the interaction between the 2 proteins are highlighted (Fig. 10M).

These findings suggest that Rh2-pre Exo regulates CCRL2 mRNA methylation through ALKBH5 and that CCRL2 can interact with TLR4 to influence the NF-κB pathway.

Finally, good biocompatibility is a necessary prerequisite for any nanomaterial to be used for drug delivery in vivo. Therefore, the biocompatibility of Rh2-pre Exo was evaluated. The results, shown in Fig. S10, indicate that Rh2-pre Exo at all concentrations did not cause hemolysis, suggesting that Rh2-pre Exo has good biocompatibility. Additionally, as shown in Fig. S11, no significant histological damage was observed in the H&E staining of heart, liver, spleen, lung, and kidney tissue sections from all treatment groups. These results further demonstrate that Rh2-pre Exo has good biocompatibility.

## Discussion

In this study, we innovatively employed Rh2-pretreated MSCs and collected their secreted exosomes. Previous research has indicated that macrophage polarization plays a crucial role in the progression of RA. Therefore, we initially focused on macrophages in vitro, demonstrating that Rh2-pre Exo can inhibit the proliferation and migration of inflammatory macrophages. We induced M1 polarization in macrophages using LPS and employed a variety of experimental techniques, including flow cytometry, immunofluorescence, qPCR, WB, and ELISA, to comprehensively validate the ability of Rh2-pre Exo to suppress M1 macrophage polarization and promote M2 conversion. We propose that this mechanism may underlie its therapeutic effects. Subsequently, we established CIA rat and mouse models to investigate the in vivo therapeutic efficacy of Rh2-pre Exo. The results demonstrated that Rh2-pre Exo substantially improved arthritis inflammation in both models. To further confirm the therapeutic effects of Rh2-pre Exo, we conducted multiple experiments on CIA mice, including H&E staining, Safranin O/Fast Green staining, x-ray imaging, micro-CT, immunohistochemistry, immunofluorescence, ELISA, WB, and small animal in vivo imaging. We found that Rh2-pre Exo could target the inflammatory sites in CIA mice, which is a prerequisite for its therapeutic effects. Furthermore, Rh2-pre Exo substantially ameliorated arthritis inflammation in CIA mice and validated its ability to inhibit M1 macrophage polarization and promote M2 conversion in animal experiments, consistent with the in vitro findings.

To explore the mechanisms by which Rh2-pre Exo alleviates arthritis in CIA mice, we employed various omics sequencing methods. Initially, we conducted proteomic sequencing on mouse synovial tissue, revealing that Rh2-pre Exo may improve arthritis inflammation in CIA mice by inhibiting NET formation and the NF-κB pathway. Subsequently, we performed MeRIP-seq and RNA-seq analyses on the synovial tissue, discovering a significant increase in m6A levels following Rh2-pre Exo treatment, mediated by the demethylase ALKBH5. The combined results of MeRIP-seq and RNA-seq confirmed that CCRL2 mRNA is a target of ALKBH5. We suggest that Rh2-pre Exo may inhibit CCRL2 expression by modulating the m6A levels of its mRNA, thereby exerting its therapeutic effects through an ALKBH5-mediated process.

Interestingly, we observed a substantial decrease in TLR4 protein expression in the synovial tissue of CIA mice after Rh2-pre Exo treatment. Through co-immunoprecipitation experiments and molecular docking techniques, we established an interaction between CCRL2 and TLR4. Given that TLR4 is an upstream regulator of the NF-κB pathway, we successfully integrated the findings from proteomic sequencing with those from MeRIP-seq and RNA-seq, leading to the conclusion that Rh2-pre Exo may regulate CCRL2 mRNA m6A levels to inhibit CCRL2 expression, subsequently impacting the TLR4/Myd88/NF-κB signaling pathway.

CCRL2 was first cloned from LPS-stimulated macrophages and was initially designated as LPS-induced C-C chemokine receptor-related gene (L-CCR) [38]. This gene encodes a 7-transmembrane protein that is closely related to CCR1 [39]. Chemokines and their receptors mediate signal transduction essential for the recruitment of effector immune cells [40]. CCRL2 is highly expressed in primary neutrophils and monocytes and is further up-regulated during neutrophil activation and the differentiation of monocytes into macrophages [41]. Studies have shown that CCRL2 mediates the migration of inflammatory cells and exerts pro-inflammatory effects in mouse models of RA and allergic asthma [41,42]. CCRL2-deficient mice exhibit impaired neutrophil recruitment, while the absence of CCRL2 in RA mice may confer protective effects [41]. Research has indicated that CCRL2 co-immunoprecipitates with Janus kinase 2 (JAK2), enhancing the JAK2–STAT (signal transducer and activator of transcription) interaction [43]. Importantly, previous studies have revealed an interaction between CCRL2 and cell surface TLR4, which is crucial for maintaining TLR4 expression on the membrane and further amplifying downstream Myd88/NF-κB inflammatory signaling in macrophages [44].

Prior research has demonstrated that activating the TLR4/Myd88/NF-κB axis can convert pro-tumor macrophages into tumor-suppressing macrophages, successfully initiating antitumor immune therapy [45]. Inhibition of the TLR4/Myd88/NF-κB pathway can reprogram macrophage polarization to an anti-inflammatory state, targeting inflamed lungs and improving acute lung injury [25,27,46]. Our study indicates that Rh2-pre Exo can suppress TLR4/Myd88/NF-κB inflammatory signaling, reprogramming macrophages to an anti-inflammatory state and thereby alleviating arthritis in CIA mice.

Emerging evidence suggests a connection between the dysregulation of N6-methyladenosine (m6A) modifications and inflammation, as well as inflammatory diseases; however, the underlying mechanisms still require further exploration. Previous research has indicated that ALKBH5-mediated RNA m6A methylation plays a role in regulating the migration, invasion, and proliferation of rheumatoid fibroblast-like synoviocytes [31]. METTL3-mediated m6A modifications enhance the stability of Hspa1a, thereby inhibiting osteocyte senescence [47]. Additionally, it has been found that elevated m6A modification levels in various high-inflammatory states depend on p65, as the key component of the “writer” complex, Wilms tumor 1 associated protein (WTAP), is transcriptionally regulated by p65, with its overexpression leading to increased m6A levels [48]. Research on WTAP has also shown that WTAP-mediated FRZB m6A modification triggers inflammatory responses via the Wnt signaling pathway [49]. These studies underscore the role of m6A modifications in inflammatory diseases. Extracellular vesicles derived from human umbilical MSCs interact with METTL3 to reduce NLRP3 m6A levels in macrophages, alleviating osteoarthritis in mouse models [50]. Furthermore, studies have found that exosomes targeting METTL14 can modulate NFATc1 m6A methylation levels to correct osteoclast-induced bone resorption [30]. These 2 studies provide insight into how m6A modification influences the therapeutic effects of exosomes in arthritis.

In this study, we elucidated how Rh2-pre Exo affects CCRL2 at the m6A level, further modulating TLR4/Myd88/NF-κB inflammatory signaling and reprogramming macrophages to an anti-inflammatory state. This may partly explain the mechanism by which Rh2-pre Exo improves arthritis in CIA mice. However, our research has certain limitations. Although this study assessed the effects of Rh2-pre Exo on the NF-κB pathway and NET formation using various techniques, further functional validation through small interfering RNA (siRNA) knockdown or inhibitor studies has not been conducted. These experiments would help clarify the specific roles of particular proteins in the action of Rh2-pre Exo. Currently, we have confirmed that Rh2-pre Exo is effectively taken up by M1 macrophages, but the intracellular targeting or transport mechanisms have not been explored in depth. Colocalization studies with cellular organelles are an important step in understanding exosome functionality. Future research will further investigate the specific pathways and roles of exosomes within cells. Overall, we have developed a promising therapeutic strategy for RA and partially elucidated its mechanisms at the m6A modification level. Compared to other exosome modification strategies, our Rh2-pre Exo approach is simpler and more effective, presenting better prospects for clinical translation.

This study elucidates the potential mechanisms by which Rh2-pretreated MSC-derived exosomes (Rh2-pre Exo) may serve as a therapeutic strategy for RA. Through both in vitro and in vivo experiments, we demonstrated that Rh2-pre Exo could inhibit the polarization of M1 macrophages while promoting the conversion to M2 macrophages, thereby alleviating arthritis symptoms. Our findings indicate that Rh2-pre Exo exerts its therapeutic effects by regulating the m6A modifications of CCRL2 mRNA, affecting the activity of the TLR4/Myd88/NF-κB signaling pathway, and subsequently reprogramming macrophages to an anti-inflammatory state. Additionally, we observed that Rh2-pre Exo could target inflammatory sites, effectively reducing inflammation in CIA mouse models.

There are certain limitations to our study. For example, miRNA sequencing of Rh2-pre Exo was not performed, nor was a comparison made with untreated MSC-Exo, and the specific mechanisms by which Rh2-pre Exo regulates ALKBH5 remain unclear. In vivo, M1 macrophages originate not only from tissue-resident macrophages but also from circulating monocytes. In the present study, we used RAW264.7 cells as an in vitro model to preliminarily investigate the effect of Rh2-pre Exo on macrophage polarization. Future studies will further explore the involvement and mechanisms of macrophages derived from circulating monocytes. Nevertheless, our study provides new insights and strategies for the treatment of RA. Compared to other exosome modification methods, Rh2-pre Exo exhibits simpler and more effective characteristics, indicating a promising potential for clinical translation. Future research should further explore its mechanisms of action to establish a more comprehensive theoretical foundation and practical guidance for the treatment of RA.

## Ethical Approval

The Animal Experimentation Ethics Committee of Jilin University approved the ethical approved projects entitled “Exploring the therapeutic potential of Rh2-pre exosomes in collagen-induced arthritis via m6A methylation modulation”. Additionally, the Ethics Committee of the China-Japan Union Hospital of Jilin University approved the human ethical aspects of this study. Informed consent was obtained from all participants, and the study adhered to the principles of the Declaration of Helsinki.

## Data Availability

All data of this study are available from the corresponding authors upon reasonable request.
