# Supplementary material for: Ginsenoside Rh2-Pretreated Mesenchymal Stem Cell Exosomes Ameliorate Collagen-Induced Arthritis via N6-Methyladenosine Methylation
Source: Biomater Res. 2025 Jun 11;29:0220. doi: 10.34133/bmr.0220 (PMC12153209; doi:10.34133/bmr.0220)
Supplement: Supplementary 1 — Table S1 Figs. S1 to S11 [file bmr.0220.f1.docx]

**Supplementary materials**

**Supplementary table 1: Primers used for qRT-PCR**

| Gene name | Primer sequence (5’-3’) |
| --- | --- |
| *Gapdh* | Forward: 5’- GGGTCCCAGCTTAGGTTCAT -3’  Reverse: 5’- CCAATACGGCCAAATCCGTT -3’ |
| *IL-1β* | Forward: 5’- ATGAAGGGCTGCTTCCAAAC -3’  Reverse: 5’- TCTCCACAGCCACAATGAGT -3’ |
| *IL-6* | Forward: 5’- GGAGCCCACCAAGAACGATA -3’  Reverse: 5’- ACCAGCATCAGTCCCAAGAA -3’ |
| *TNF-α* | Forward: 5’- CTCATGCACCACCATCAAGG -3’  Reverse: 5’- ACCTGACCACTCTCCCTTTG -3’ |
| *IL-10* | Forward: 5’- CTGGACAACATACTGCTAACCG -3’  Reverse: 5’- GGGCATCACTTCTACCAGGTAA -3’ |
| *iNOS* | Forward: 5’- GAGACAGGGAAGTCTGAAGCAC -3’  Reverse: 5’- CCAGCAGTAGTTGCTCCTCTTC -3’ |
| *Arg-1* | Forward: 5’- TGGCTTGCGAGACGTAGAC -3’  Reverse: 5’- GCTCAGGTGAATCGGCCTTT -3’ |
| *Mettl3* | Forward: 5’- CTGGGCACTTGGATTTAAGGAA -3’  Reverse: 5’- TGAGAGGTGGTGTAGCAACTT -3’ |
| *Mettl14* | Forward: 5’- CTGAGAGTGCGGATAGCATTG -3’  Reverse: 5’- GAGCAGATGTATCATAGGAAGCC -3’ |
| *Alkbh5* | Forward: 5’- CGCGGTCATCAACGACTACC -3’  Reverse: 5’- ATGGGCTTGAACTGGAACTTG -3’ |
| *Fto* | Forward: 5’- TTCATGCTGGATGACCTCAATG -3’  Reverse: 5’- GCCAACTGACAGCGTTCTAAG -3’ |
| *Wtap* | Forward: 5’- GAACCTCTTCCTAAAAAGGTCCG -3’  Reverse: 5’- TTAACTCATCCCGTGCCATAAC -3’ |
| *Ccrl2* | Forward: 5’- GCCCCGGACGATGAATATGAT -3’  Reverse: 5’- CACCAAGATAAACACCGCCAG -3’ |
| *Rab44* | Forward: 5’- CCCAGGGCAAATAAAGAGGCT -3’  Reverse: 5’- AGAGGTGCTACTCGGGCTT -3’ |
| *Disp3* | Forward: 5’- CCGGAGGGTCAGGTAACCA -3’  Reverse: 5’- CTCACATAGGTGCGCGAGTAA -3’ |
| *Tspoap1* | Forward: 5’- CCAAGCATTGCTGGTACTCC -3’  Reverse: 5’- GACAGGTCCAGGGCTATGAG -3’ |
| *Rdm1* | Forward: 5’- CACCCAGAGCGACAAAGTTTT -3’  Reverse: 5’- GGGAAGACTCGGACTGAATACA -3’ |
| *Fmn2* | Forward: 5’- ATGGGGAACCAGGATGGGAA -3’  Reverse: 5’- CTTGCCAAGCGCCTTTTTG -3’ |
| *Wdr54* | Forward: 5’- CTCCATCCCACTTCGTAGCTC -3’  Reverse: 5’- GGTCCATGAACCACCCCAA -3’ |
| *Rnf112* | Forward: 5’- TGGATCTCTTAGTCCGAGACTCT -3’  Reverse: 5’- GCTTCTGGAGTATGTCACCCAC -3’ |
| *Wscd2* | Forward: 5’- CAGCGGTGTTTCCGGTATTTC -3’  Reverse: 5’- CAGGTGCAAATCACCCAGGA -3’ |
| *Dzip1l* | Forward: 5’- CTCGCCGTGAAAGCATAGACT -3’  Reverse: 5’- CCGTCCAGGTTACAGAAGGT -3’ |


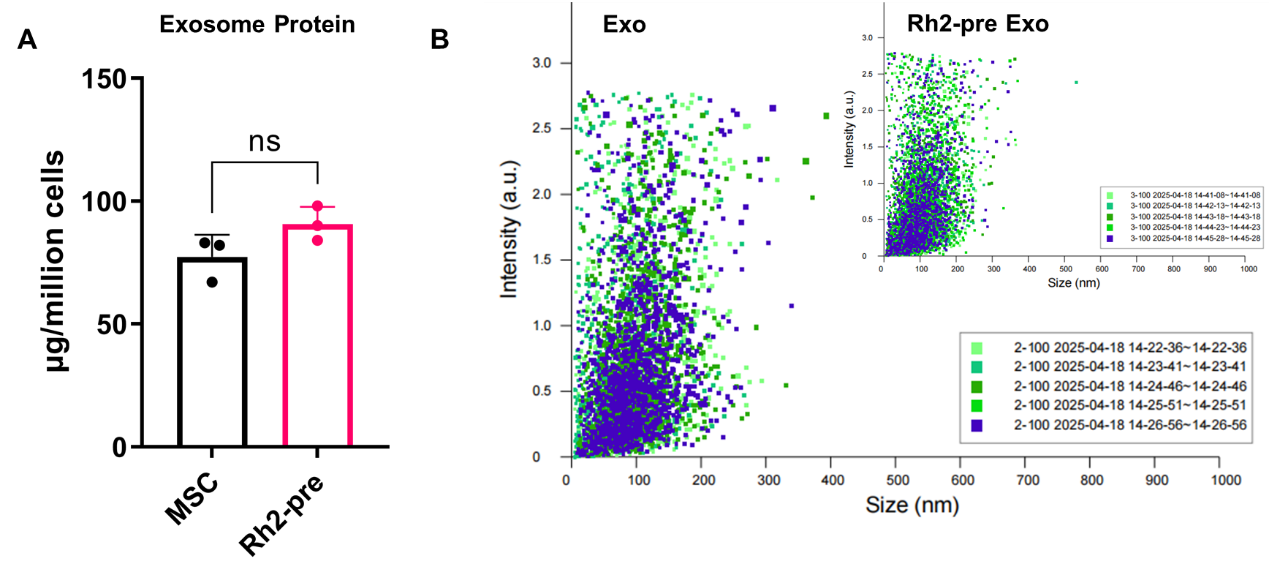


**Supplementary figure 1.** **(A)**The protein content of exosomes secreted by normal MSCs and Rh2-pretreated MSCs was measured using a BCA assay. **(B)**Fluorescence intensity was used to quantify the size distribution of Exo and Rh2-pre Exo.


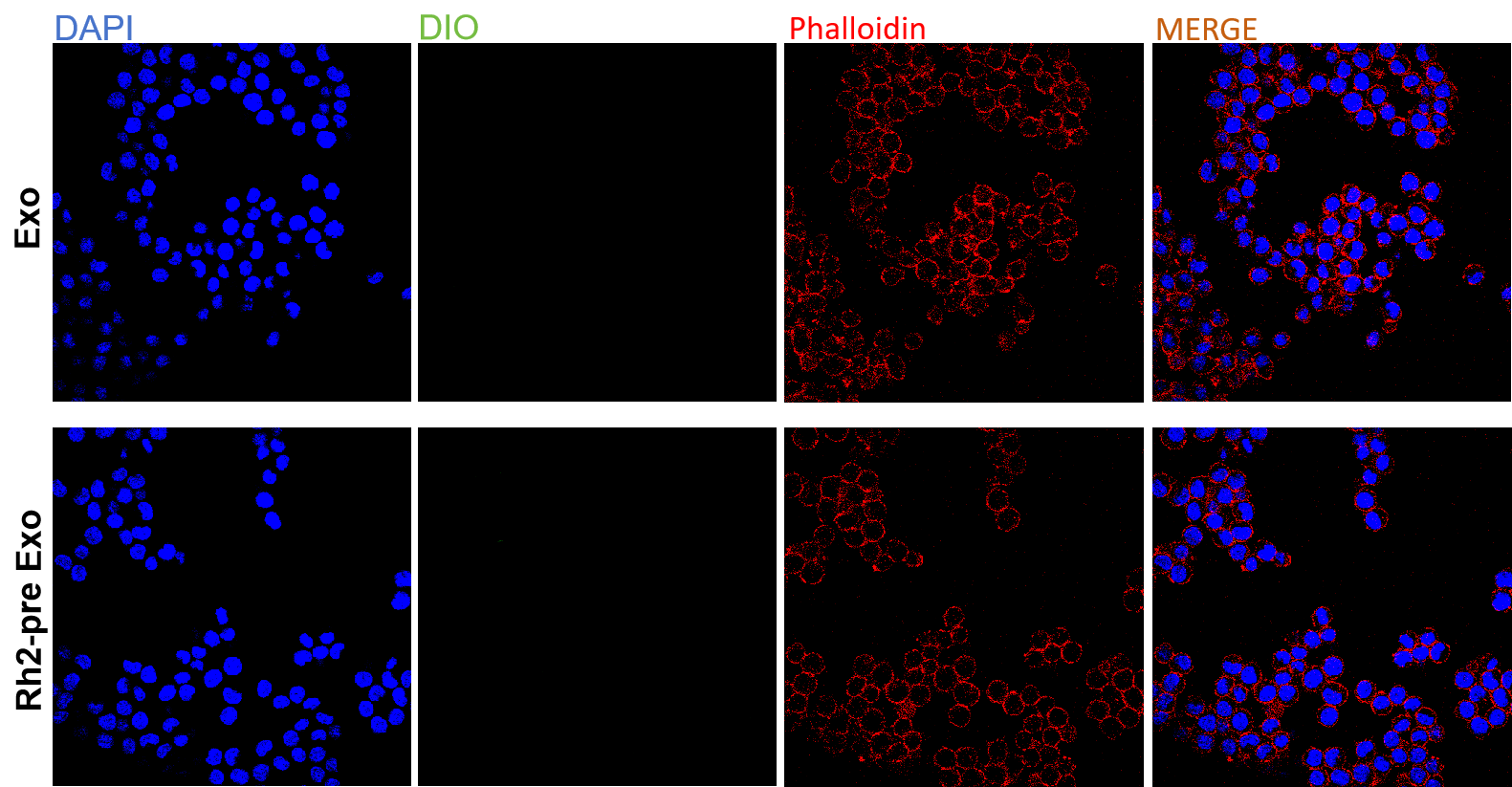


**Supplementary figure 2.** **The experimental results of exosome uptake by RAW264.7 cells without LPS stimulation.** The green fluorescence represents exosomes stained with DIO, the orange-red fluorescence represents the cytoskeleton stained with SF555-labeled phalloidin, and the blue fluorescence represents the cell nuclei stained with DAPI. Scale bar = 50 µm.


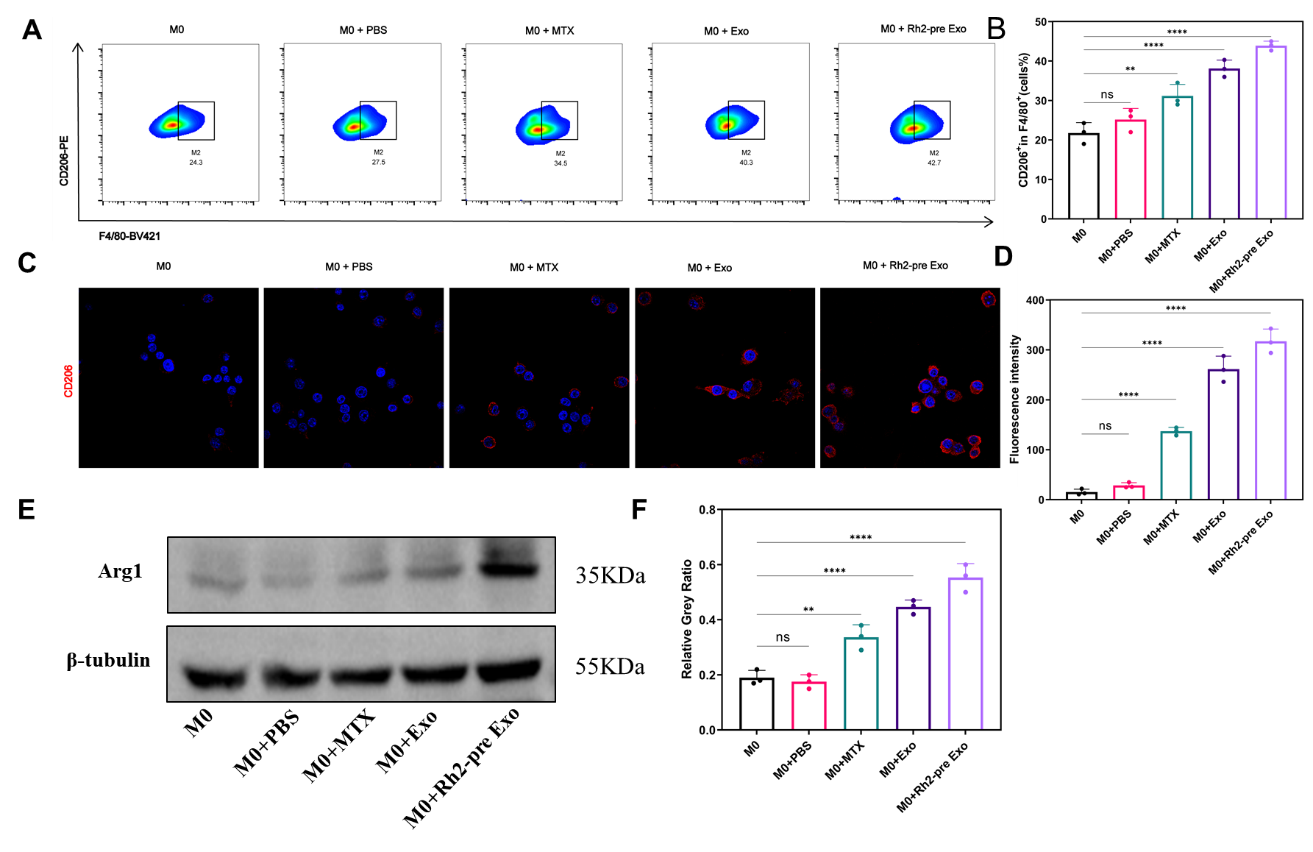


***Supplementary figure 3. Rh2-pre Exo promotes the polarization of monocyte-derived macrophages toward the M2 phenotype.*** We treated THP-1 macrophages in each group with 10 μg/mL of EVs for 24 hours, after which cells were immediately collected for subsequent analyses. (A) Flow cytometry results of CD206 expression. (B) Bar chart of CD206 expression analyzed by flow cytometry. N=3. (C) Results of immunofluorescence staining for CD206. Scale bar = 30 μm. (D) The bar chart displays the statistical results of the fluorescence intensity of CD206 in different groups. N=3. (E) Western blot (WB) analysis was conducted to examine the protein expression levels of Arg1 in THP-1 cells. N=3. (F) Bar graphs were generated to quantify the grayscale analysis of Arg1 protein expression. N=3. **p<0.01, ****p<0.0001.


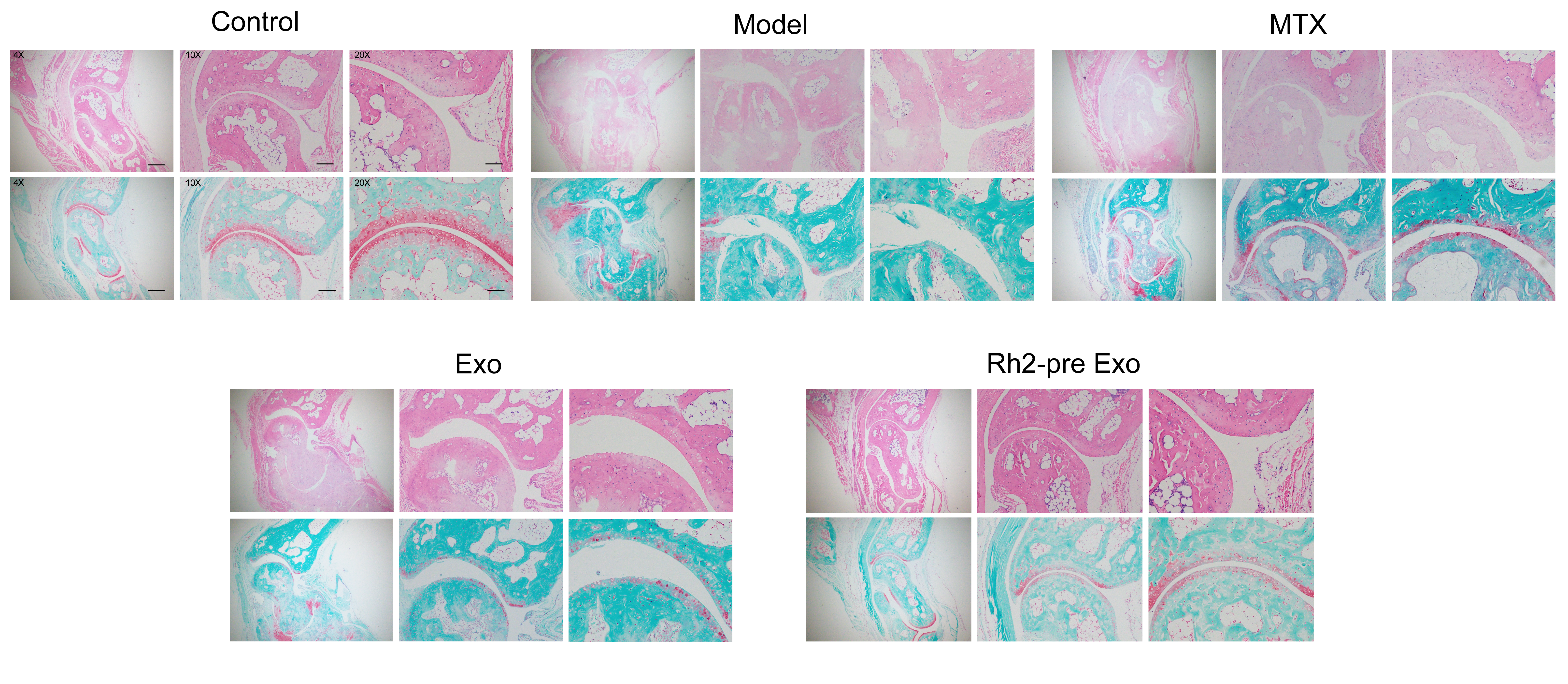


**Supplementary figure 4.** **H&E Staining and Safranin O/Fast Green Staining Results**: Histological analysis of ankle joints from different treatment groups, with scale bars indicating (4x) = 500 µm, (10x) = 200 µm, and (20x) = 100 µm.


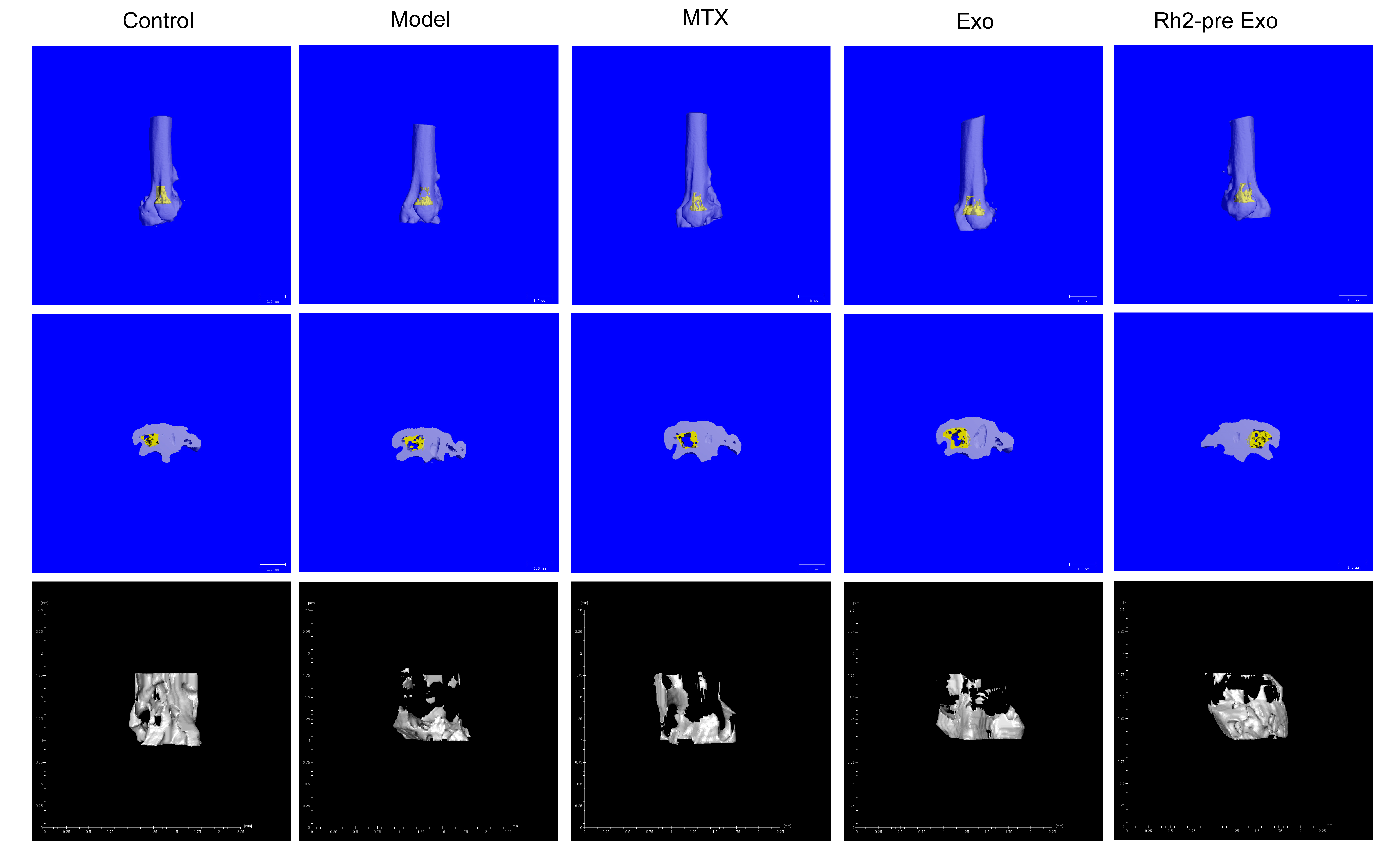


**Supplementary figure 5. 3D Imaging of Distal Tibia:** 3D reconstruction of specific regions in the distal tibia of mice from different treatment groups. The region of interest (ROI) was defined by a trabecular height of 0.9 mm on the 2D image, followed by thresholding at Threshoid = 200 for data analysis.


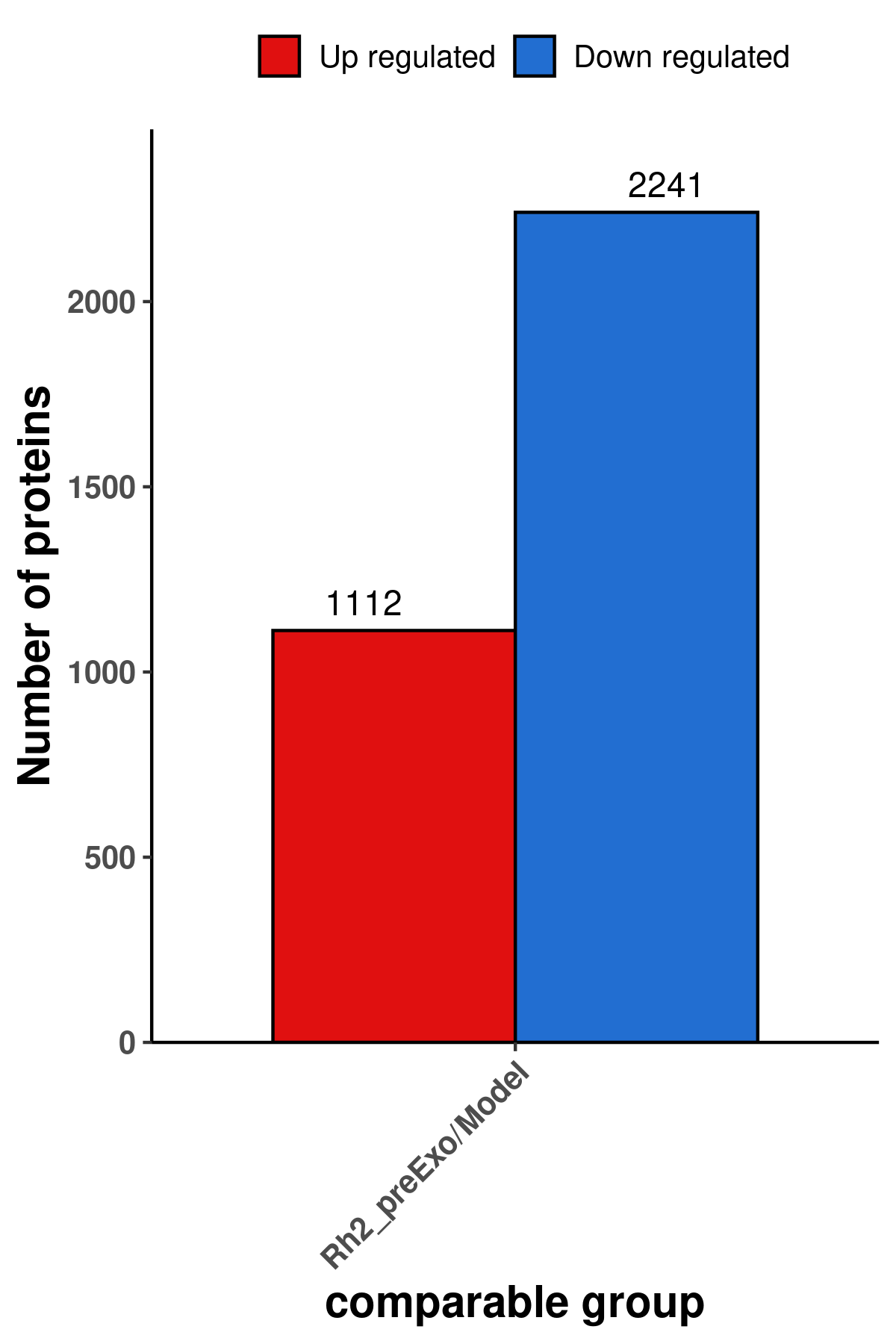


**Supplementary figure 6.** **Number of Differentially Expressed Proteins**

**
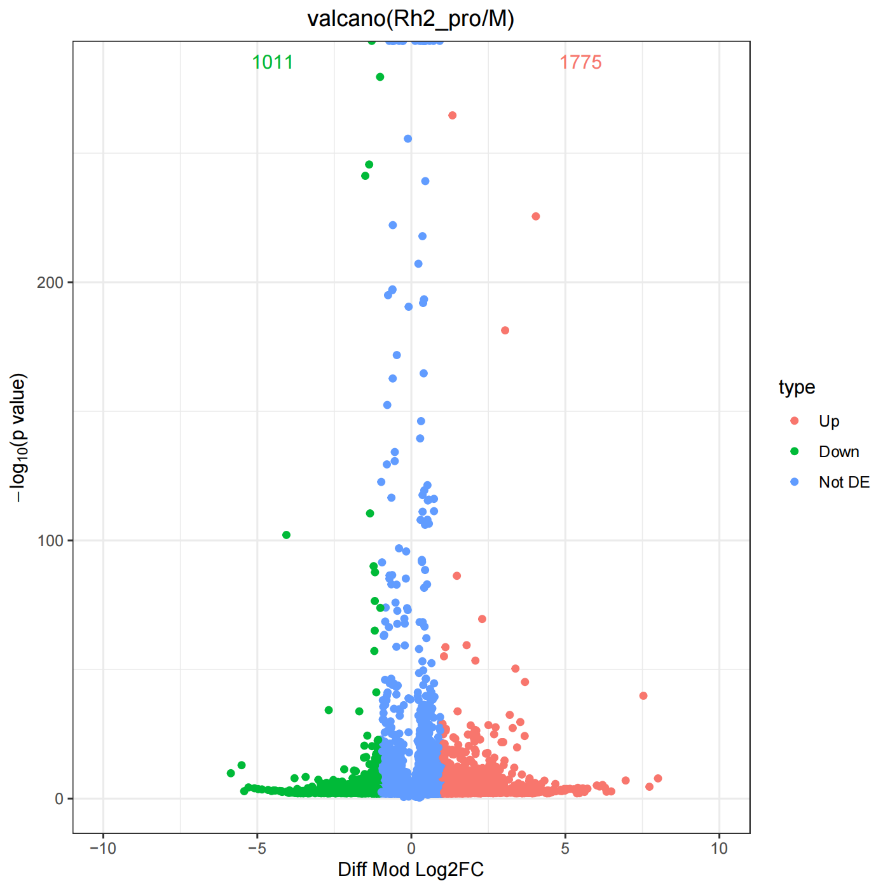
**

**Supplementary figure 7. Differential Peak Volcano Plot:** Volcano plot comparing peaks between the model and Rh2-pre Exo treatment groups. Red points indicate upregulated peaks in case samples compared to controls, green points indicate downregulated peaks, and blue points represent peaks with no significant difference. The vertical axis shows the p-value, where smaller values indicate greater significance, leading to higher -log10(p-value) values. Points in the upper left and upper right corners represent highly significant downregulated and upregulated peaks, respectively.


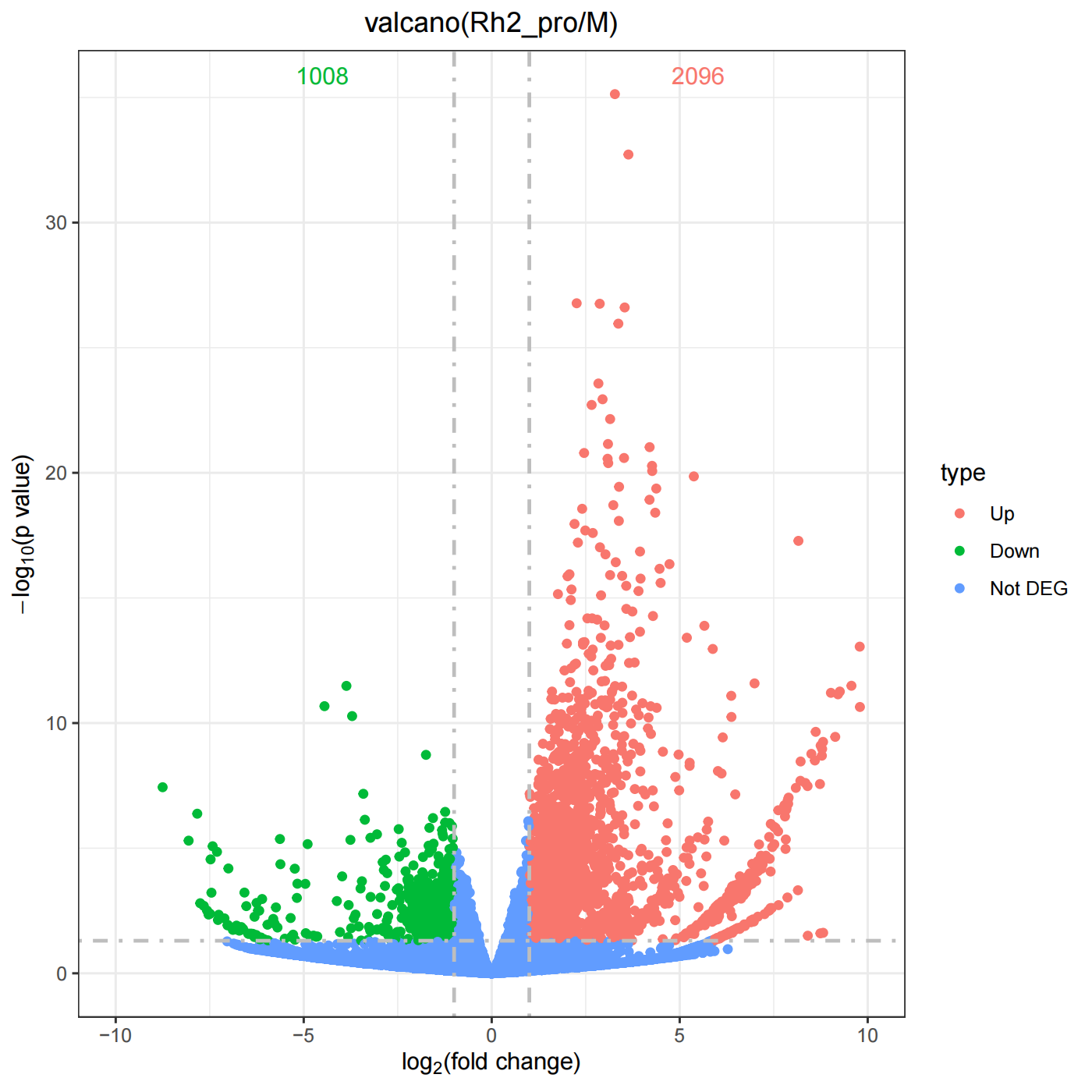


**Supplementary figure 8.** **Differential mRNA Expression Volcano Plot:** Volcano plot displaying mRNA expression differences between the model and Rh2-pre Exo treatment groups. Red points represent upregulated genes in case samples compared to controls, green points represent downregulated genes, and blue points indicate genes with no significant difference. The vertical axis shows the p-value, with interpretation as above for peaks.

**
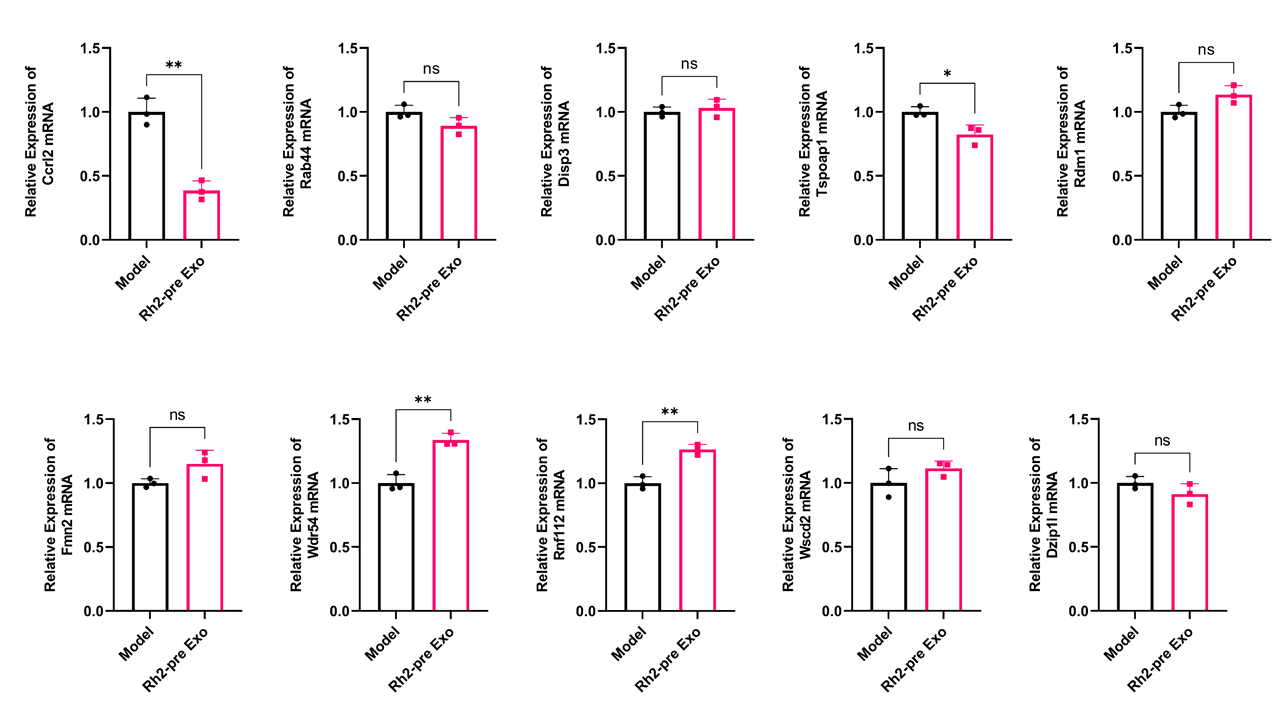
**

**Supplementary figure 9. qPCR Validation of Gene Expression:** qPCR was performed to validate the expression of genes Ccrl2, Rab44, Disp3, Tspoap1, Rdm1, Fmn2, Wdr54, Rnf112, Wscd2, and Dzip1l in the model and Rh2-pre Exo treatment groups. N = 3.


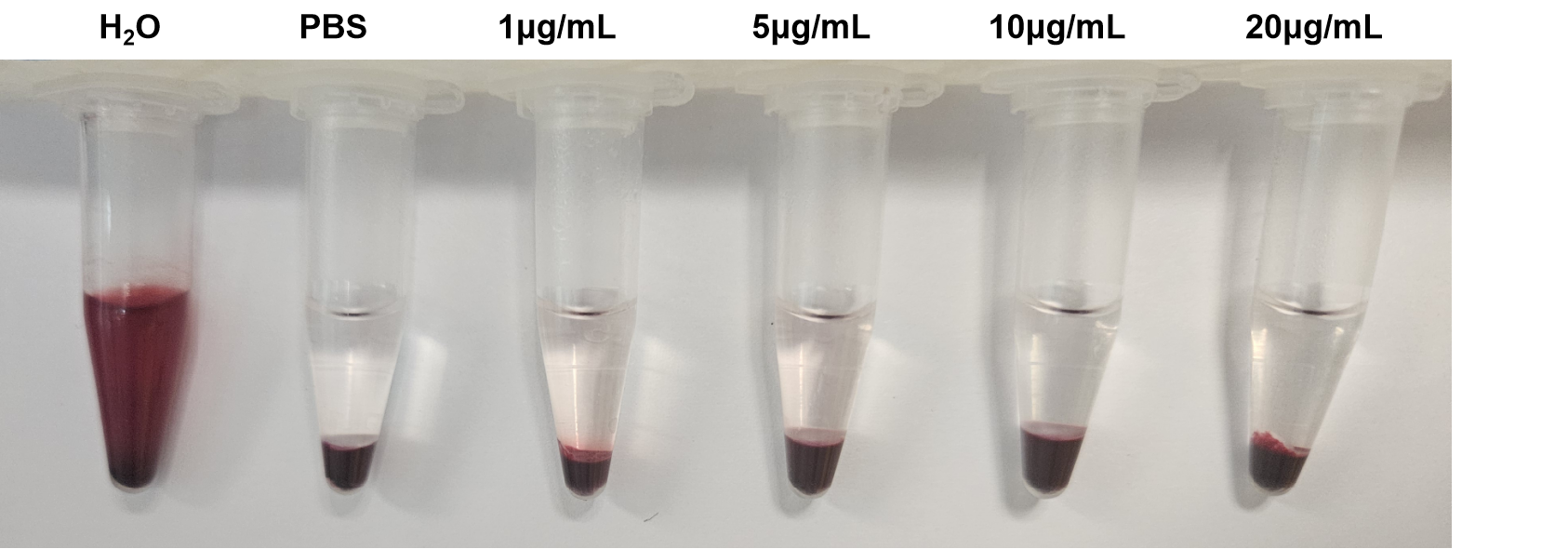


**Supplementary figure 10. The hemolysis assay results of Rh2-pre Exo.**


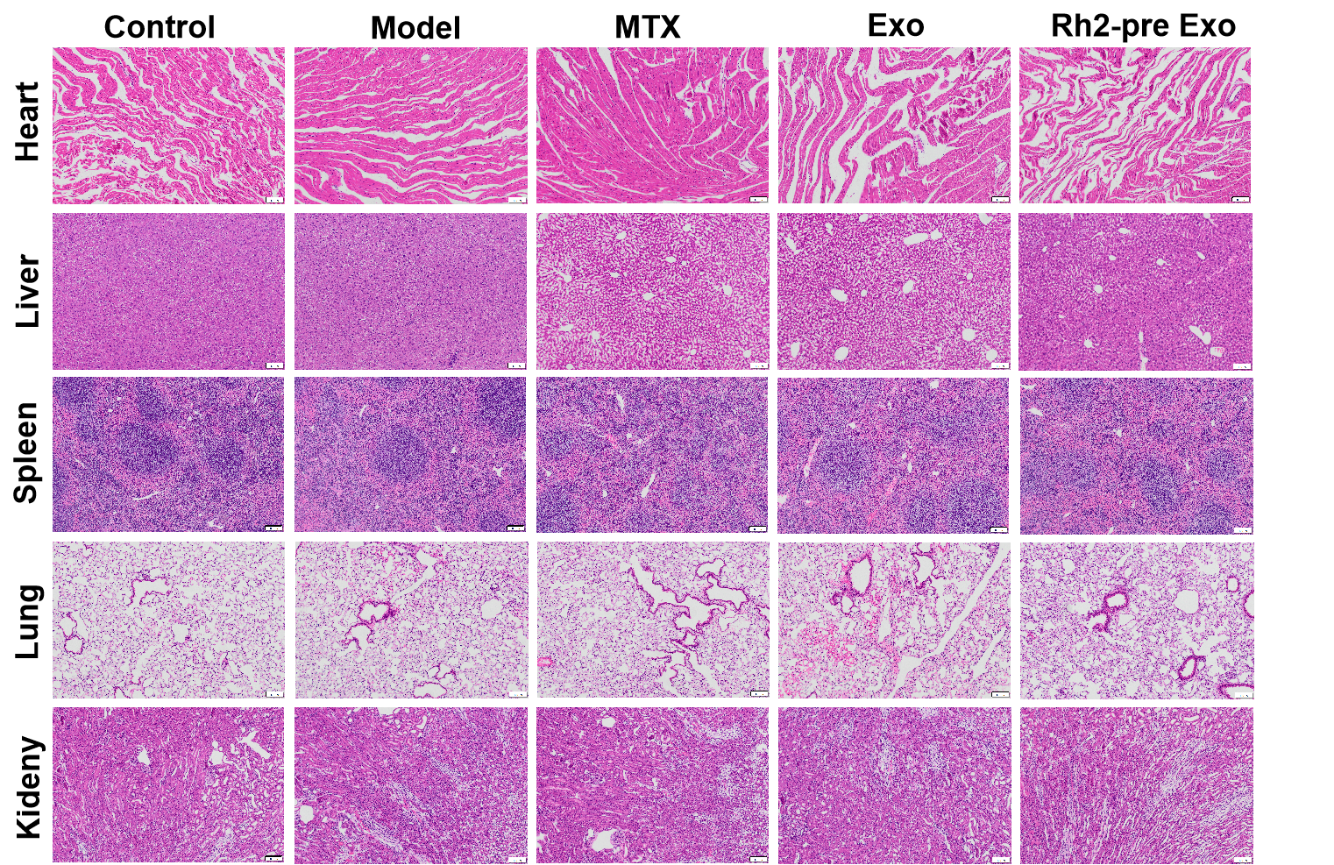


**Supplementary figure 11. H&E staining and light microscopy observation of the heart, liver, spleen, lung, and kidney from mice in each group (scale bar: 100 μm).**
